# Two Species with an Unusual Combination of Traits Dominate Responses of British Grasshoppers and Crickets to Environmental Change

**DOI:** 10.1371/journal.pone.0130488

**Published:** 2015-06-25

**Authors:** Björn C. Beckmann, Bethan V. Purse, David B. Roy, Helen E. Roy, Peter G. Sutton, Chris D. Thomas

**Affiliations:** 1 Centre for Ecology & Hydrology, Wallingford, Oxfordshire, United Kingdom; 2 Department of Biology, University of York, York, United Kingdom; 3 Orthoptera and Allied Insects Recording Scheme of Britain and Ireland, c/o Biological Records Centre, Centre for Ecology & Hydrology, Wallingford, Oxfordshire, United Kingdom; Muséum national d'Histoire naturelle, FRANCE

## Abstract

There are large variations in the responses of species to the environmental changes of recent decades, heightening interest in whether their traits may explain inter-specific differences in range expansions and contractions. Using a long-term distributional dataset, we calculated range changes of grasshoppers and crickets in Britain between the 1980s and the 2000s and assessed whether their traits (resource use, life history, dispersal ability, geographic location) explain relative performance of different species. Our analysis showed large changes in the distributions of some species, and we found a positive relationship between three traits and range change: ranges tended to increase for habitat generalists, species that oviposit in the vegetation above ground, and for those with a southerly distribution. These findings accord well with the nature of environmental changes over this period (climatic warming; reductions in the diversity and increases in the height of vegetation). However, the trait effects applied mainly to just two species, *Conocephalus discolor *and *Metrioptera roeselii*, which had shown the greatest range increases. Once they were omitted from the analysis, trait effects were no longer statistically significant. Previous studies on these two species emphasised wing-length dimorphism as the key to their success, resulting in a high phenotypic plasticity of dispersal and evolutionary-ecological feedback at their expanding range margins. This, combined with our results, suggests that an unusual combination of traits have enabled these two species to undertake extremely rapid responses to recent environmental changes. The fact that our results are dominated by two species only became apparent through cautious testing of the results’ robustness, not through standard statistical checks. We conclude that trait-based analyses may contribute to the assessment of species responses to environmental change and provide insights into underlying mechanisms, but results need to be interpreted with caution and may have limited predictive power.

## Introduction

The responses of individual species to environmental change are highly variable, despite average polewards and upwards range shifts of species responding to climate change, and contractions of species ranges in regions experiencing habitat loss, degradation and fragmentation [1–5]. Often, different environmental changes interact to affect species and result in a wide range of responses [6, 7]. At present, we have limited ability to predict the attributes of species that will thrive and exploit new opportunities, and of those that will decline and fail to adapt to changing conditions [8, 9]. Understanding this variation represents a fundamental scientific challenge, the answers to which will have relevance to the conservation of species and species communities, and to the wider management of ecosystems.

The natural environment has been subject to extensive changes over recent decades. Global average surface air temperatures have risen by about 0.8°C since 1900, much of this rise occurring in the past 30 to 40 years, making the speed of recent warming faster than most past climatic changes [10]. The mean Central England Temperature in the 2000s was 0.84°C higher than in the 1980s [11]. Globally, conversion of natural habitats to agriculture reached an unprecedented rate in the second half of the 20^th^ century and this continues in most parts of the world. In contrast, in many developed countries conversion to agricultural use has slowed or stabilized [12]. In England, large areas of land were taken out of production as “set aside” from 1990 onwards, as a result of farming subsidies, and soon exceeded 10% of arable land, remaining at around this level until payments were stopped from 2008 [13]. However, agricultural practices continue to intensify at a global scale; nitrogen fixation through human activity, mainly fertilizer production, now equals or exceeds fixation in natural ecosystems, and considerable proportions are lost to the environment [14]. Large areas of the UK exceed “critical loads” of nutrient nitrogen, i.e. levels harmful to sensitive elements of the environment [15]. Such climatic and land use changes may strongly affect species communities and the matrix of habitats available; for example in Britain average plant species richness has decreased across habitats since 1978, with light-loving species of shorter turf declining and competitive species of fertile ground increasing [16].

We used grasshoppers and crickets (Orthoptera) as a model group to study the impacts of these changes and to identify traits which explain why species may vary in the extent to which their geographic ranges are changing. Grasshoppers and crickets are a suitable group because they are ectothermic insects found predominantly in open habitats, and are consequently highly responsive to climatic and land use changes [17–20]. In addition, the species in Britain display a broad range of biological traits [21], which might underpin their different distribution changes [8]. Many grasshoppers and crickets are easily observed and identified, and the “Orthoptera Recording Scheme” has produced a large dataset which is available for research [22]. Trait-based analyses have previously been carried out on temperate grasshoppers and relatives, including investigations of range sizes [17], extinctions [23], degree of nestedness [24], species richness [25, 26] and community composition [27], but none across species at a national scale with a focus on investigating range change.

We considered here a series of traits that might be expected to influence the responses of species to a variety of land use and climatic changes:

### Resource use traits

Under conditions of environmental change, generalists that are capable of exploiting a wide range of resources are more likely to be able to survive changes to the availability of a specific resource in a landscape, and they are more likely than specialists to be able to exploit new landscapes if climatic or other conditions become suitable [28, 29]. Numbers of habitats exploited and diet are commonly used as measures for the degree of species’ resource specialisation [30, 31]. Preferred vegetation structures and oviposition sites are two further traits that may describe species’ resource requirements, because they play a critical role in determining the suitability of habitats in terms of microclimatic conditions, particularly for ectothermic insects [26, 32–35]. We hypothesised that range changes would be positively related to traits indicative of microclimatic requirements favoured by recent land use change, i.e. a preference for medium or tall vegetation, and oviposition above ground.

### Life history traits

Species’ life history traits influence their rates of reproduction, and hence exert an important influence on their ability to respond to environmental change; for example the number of generations per year and body size [8, 36]. We predicted that the greatest range increases would be positively related to traits associated with fast reproduction, i.e. short generations, and small body size. Winter stage (i.e. life stage at which the species overwinters) and phenology (i.e. seasonal timing of the life cycle) may influence species’ vulnerability to adverse weather and their ability to exploit favourable seasonal conditions [37–39]. For a group of species with the same over-winter stage, those that mature later in the season are likely to have a greater degree of “thermal limitation”, i.e. to require a greater sum of warmth for development. We therefore predicted late-maturing species to have increased their ranges more, since they would be likely to benefit more from recent climatic warming.

### Traits characterising dispersal ability

A critical factor determining species’ capacity to respond to environmental change is their dispersal ability, particularly if the rate of that change is rapid [18, 25, 28], although if habitat is highly fragmented selection may also act against dispersal [40]. Wing length and wing load are species traits commonly used to approximate dispersal ability in insects [41]. We predicted that increases in range would be positively related to wing morphology favouring dispersal—i.e. long wings or wing-length dimorphism, and low wing load. British grasshoppers and crickets include several species which exhibit wing dimorphism, with a short-winged (brachypterous) form and a long-winged, particularly dispersive (macropterous), form. Strong trade-offs between investment in the flight apparatus and investment in reproductive organs mean that wing-dimorphic species may be at a selective advantage by producing increased numbers of macropterous individuals only under conditions favouring dispersal [18, 27]. Therefore, wing-dimorphic species were predicted to have shown more positive distribution trends over the recent decades of environmental change than obligate macropters or obligate brachypters.

### Distributional traits

Parameters of species’ distributions including average latitude or position of distributional margins have been used as measures of their climatic requirements [5]. Species with lower average latitudes are likely to be more thermally limited than those with higher ones and hence to benefit more from warming; we therefore predicted a negative relationship between average latitude and range change.

Using these biological traits and hypotheses, we assessed their relative importance in explaining distributional changes of grasshoppers and crickets in Britain between the 1980s and 2000s.

## Materials and Methods

### Range changes

The extent of changes in distributions of British grasshoppers and crickets was quantified using the data of the Orthoptera Recording Scheme [22, 42, 43]. The scheme has collated 104,144 distribution records from over 2,000 volunteers since 1967. Records are mostly gathered in a non-standardised way, i.e. with no standard protocol or measure of recording effort, the main aim being to record distributions. Locations are recorded to varying degrees of precision, many to a 100m grid square resolution or finer (55%), with the rest at 1km, 2km or 10km resolutions (27%, 3% and 15% respectively). Data were summarised at a 10km grid square (“hectad”) resolution, based on the British National Grid, and the analysis was restricted to the mainland and inshore islands of Great Britain (England, Scotland, Wales). All calculations were performed in the statistical software environment “R”, version 3.0.2 [44].

Changes in species range sizes were calculated between the decades 1980–89 and 2000–09. These periods were selected to cover the time of most intense recording and therefore to maximise the number of records available for analysis while maintaining a gap between them. The periods were also selected to cover a time of extensive environmental change both in climate and land use (mean Central England Temperature increasing by 0.84°C; in excess of 10% of arable land taken out of cultivation; and nutrient enrichment of many habitats continuing—resulting in vegetation becoming taller, more shaded and less diverse [11, 13, 15, 16]; cf. Introduction and Discussion).

**Fig 1:**
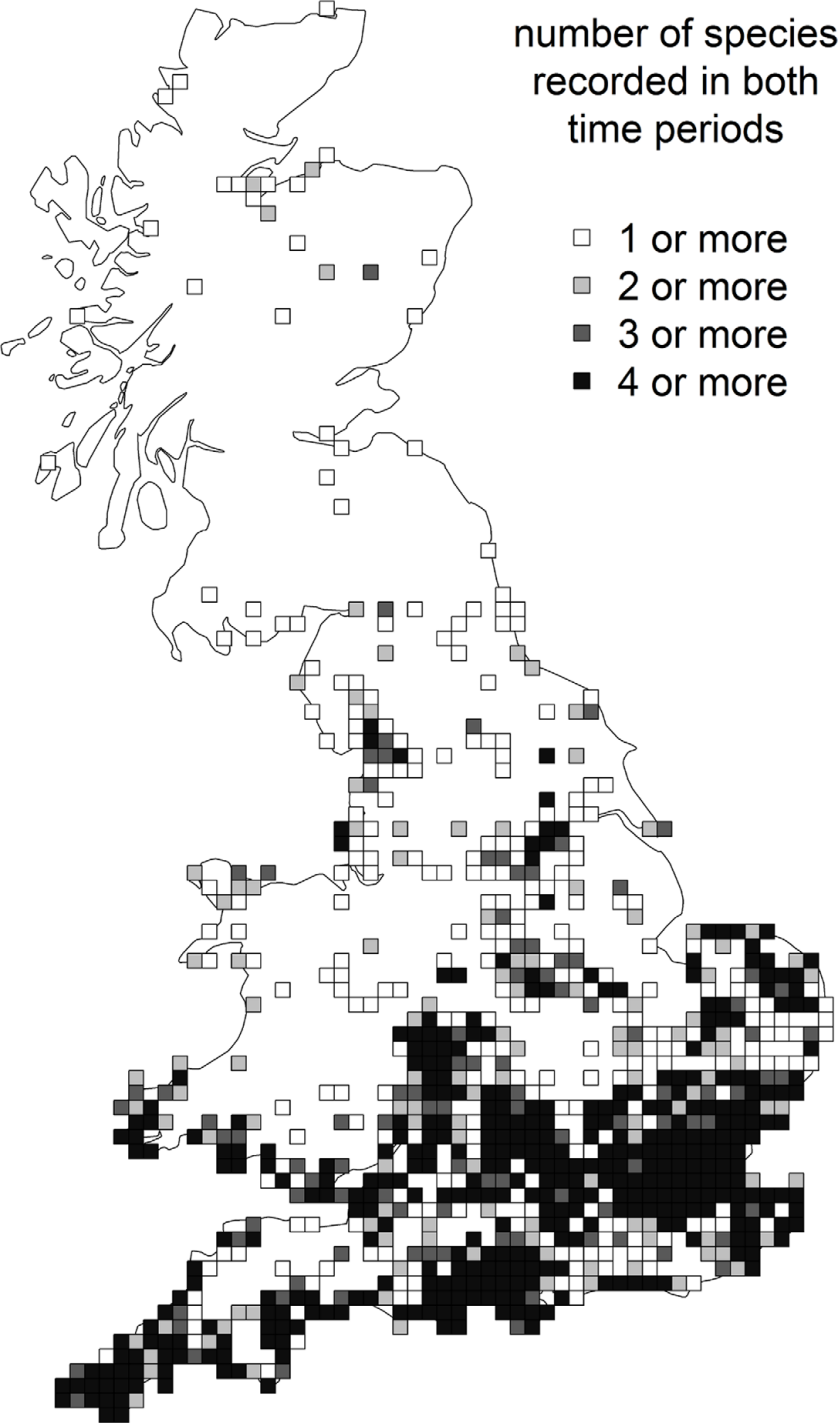
Location of four sets of “surveyed squares” with different levels of recording effort. 10km grid squares on the British mainland and inner islands with respectively at least one, two, three or four grasshopper or related species recorded in both the periods 1980–9 and 2000–9. There was a total of 844 squares with at least one species recorded in both time periods (32% of the possible total of 2,662 squares), 598 squares (22%) with at least two species, 474 squares (18%) with at least three, and 375 squares (14%) with at least four.

Range changes were calculated from grid cells that had been surveyed in both time periods in order to minimise any effect of differences in the number of grid cells visited or the geographical pattern of recording. To understand impacts of increasing recorder effort on range change measures, four sets of these “surveyed squares” were defined: hectads with a minimum, respectively, of one, two, three or four grasshopper or cricket species recorded in both time periods (these were not necessarily the same species in both periods) (Fig 1, cf. [5]). Range change measures were calculated for each of these four sets of “surveyed squares” / levels of recording effort, and Pearson’s correlation tests carried out between them in order to assess their consistency. For all levels of recording effort, the majority of “surveyed squares” were located in the southern half of England with lower numbers in northern England, Wales and Scotland; this is not surprising as it reflects grasshopper and related species diversity as well as human population (and hence recorder) density, but it should be borne in mind when interpreting results.

Species range changes were calculated in two ways: (1) “Uncorrected range change” was defined simply as the absolute difference between the (logit-transformed) proportion of “surveyed squares” occupied by each species in the 2000s vs. the 1980s. Proportions were logit-transformed in order to create unbounded distributions and help to achieve normality [45]. (2) “Corrected range change”: The dataset showed an approximate doubling of recording effort between the 1980s (13,188 records for the species investigated here) and the 2000s (26,239 records). We therefore calculated a relative range change index which measured the difference in the observed range change of each species relative to the mean observed change for the whole taxonomic group, thus accounting for overall changes in recording effort (albeit at the cost of providing a purely relative measure) [46]. The index was calculated by fitting a linear regression of the logit-transformed proportions of “surveyed squares” occupied by species in the 2000s vs. the 1980s; the standardised residuals of this regression were defined as the relative index. This “corrected range change” (“Telfer”) has been shown to be robust to multiple potential biases in recording, if rather conservative [47]. In order to further check that observed range changes were genuine and not influenced unduly by large one-off population fluctuations we plotted annual relative numbers of hectads recorded per species over the entire study period 1980–2009 for species with large range change values.

Grasshopper, cricket and bush-cricket species native to Britain were included in the analyses; species occupying fewer than five hectads in the 1980s were excluded, because for the rarest species small changes in distribution or recording may affect trend calculations disproportionately [46]. This left a total of 23 species for the present study (Table 1).

**Table 1:** Species traits, range sizes, and “uncorrected range change” and “corrected range change” values.

species		habitat and resource use				life history				dispersal ability		distri-bution	raw grid square counts, percentage changes & range change measures (based on “surveyed squares” with at least 1 species recorded)					
Scientific name	English name	breadth of habitat use	preferred vegetation structure (short S, medium M, tall T)	oviposition site (ground G, ground or vegetation GV, vegetation V)	diet (herbivorous H, not herbivorous not_H)	mean body size (mm)	number of generations per year (one O, half H, half or one HO)	winter stage (egg E, larva or adult not_E)	phenology: month quarter of first appearance of adults	wing morph (short S, long L, dimorphic D)	wing load	average latitude	range size 1980–9 (no. of "surveyed squares" occupied)	range size 2000–9 (no. of "surveyed squares" occupied)	% change in range size	"uncorrected range change"	"corrected range change", all species	"corrected range change", excluding *C*. *discolor* and *M*. *roeselii*
		(i)	(ii)	(iii)	(iv)	(v)	(vi)	(vii)	(viii)	(ix)	(x)	(xi)						
*Meconema thalassinum*	Oak bush-cricket	4	T	V	not_H	15.0	HO	E	7.75	L	0.043	51.70	315	294	-7	-0.11	-0.34	-0.41
*Tettigonia viridissima*	Great green bush-cricket	3	M	G	not_H	32.5	H	E	7.5	L	0.043	51.01	139	125	-10	-0.13	-0.43	-0.39
*Pholidoptera griseoaptera*	Dark bush-cricket	5	T	V	not_H	17.5	HO	E	7.5	S	0.001	51.45	405	401	-1	-0.02	-0.19	-0.18
*Platycleis albopunctata*	Grey bush-cricket	2	M	GV	not_H	20.0	O	E	7.25	L	0.038	50.67	52	49	-6	-0.06	-0.43	-0.16
*Metrioptera brachyptera*	Bog bush-cricket	1	M	V	not_H	15.0	H	E	7.5	D	0.107	51.69	74	68	-8	-0.09	-0.44	-0.26
*Metrioptera roeselii*	Roesel’s bush-cricket	6	M	V	not_H	16.5	HO	E	6.75	D	0.118	51.65	71	332	+368	1.95	2.31	
*Conocephalus discolor*	Long-winged conehead	10	M	V	not_H	19.0	O	E	7.75	D	0.042	50.82	46	378	+722	2.63	3.23	
*Conocephalus dorsalis*	Short-winged conehead	6	M	V	not_H	14.5	O	E	7.75	D	0.089	51.50	137	213	+55	0.55	0.48	1.53
*Leptophyes punctatissima*	Speckled bush-cricket	4	T	V	H	13.5	HO	E	7.75	S	0.001	51.41	337	424	+26	0.42	0.39	1.10
*Nemobius sylvestris*	Wood cricket	2	T	G	not_H	8.5	H	not_E	6.0	S	0.015	50.82	18	19	+6	0.05	-0.36	0.24
*Tetrix ceperoi*	Cepero’s groundhopper	5	S	GV	H	9.0	O	not_E	5.0	L	0.130	50.84	25	27	+8	0.08	-0.30	0.29
*Tetrix subulata*	Slender groundhopper	7	S	GV	H	10.0	O	not_E	4.25	D	0.127	51.54	171	282	+65	0.68	0.67	1.87
*Tetrix undulata*	Common groundhopper	9	S	GV	H	9.0	O	not_E	3.75	D	0.137	51.78	309	298	-4	-0.06	-0.27	-0.26
*Stethophyma grossum*	Large marsh grasshopper	2	M	GV	H	27.0	O	E	7.75	L	0.022	50.89	14	7	-50	-0.67	-1.40	-1.90
*Stenobothrus lineatus*	Stripe-winged grasshopper	4	S	GV	H	20.5	O	E	6.25	L	0.020	51.30	68	72	+6	0.06	-0.24	0.18
*Omocestus rufipes*	Woodland grasshopper	1	M	G	H	16.5	O	E	6.0	L	0.035	50.97	49	37	-24	-0.29	-0.74	-0.81
*Omocestus viridulus*	Common green grasshopper	8	M	GV	H	18.5	O	E	5.75	L	0.027	52.25	402	350	-13	-0.25	-0.51	-0.85
*Chorthippus brunneus*	Field grasshopper	7	S	G	H	19.0	O	E	5.75	L	0.033	51.90	553	500	-10	-0.27	-0.50	-0.98
*Chorthippus vagans*	Heath grasshopper	3	M	G	H	17.0	O	E	7.25	L	0.027	50.75	6	6	0	0.00	-0.54	0.14
*Chorthippus parallelus*	Meadow grasshopper	14	M	G	H	18.0	O	E	6.0	D	0.034	51.73	526	503	-4	-0.11	-0.29	-0.50
*Chorthippus albomarginatus*	Lesser marsh grasshopper	6	M	GV	H	18.0	O	E	7.0	L	0.025	51.70	123	241	+96	0.85	0.87	2.37
*Gomphocerippus rufus*	Rufous grasshopper	5	M	G	H	19.0	O	E	7.75	L	0.023	51.23	27	25	-7	-0.08	-0.51	-0.17
*Myrmeleotettix maculatus*	Mottled grasshopper	7	S	G	H	14.0	O	E	5.75	L	0.040	52.15	206	157	-24	-0.34	-0.70	-1.04

For definitions of traits see Table 2, for details of calculation of range change measures see text.

### Species traits

A database of British grasshopper and related species traits covering habitat and resource use, life history, dispersal ability, and distribution was compiled to address the hypotheses of factors affecting range change outlined in the introduction (Tables 1 and 2).

**Table 2:** Definitions of species traits and sources of information.

	trait		definition	source
habitat and resource use	(i)	*breadth of habitat use*	total number of habitat types known per species (mean ± s.d. = 5.3 ± 3.1); log-transformed	summary table of habitats in [48]
	(ii)	*preferred vegetation structure*	typical vegetation height of species’ habitats: “Short”: open ground, short vegetation < = 20cm (6 species). “Medium”: medium or long herbaceous vegetation >20cm, patchy, early succession scrub (13 species). “Tall”: woodland, trees, hedgerows and medium or late succession scrub (4 species).	“habitat” sections of species accounts in [21]; the categories in the present study summarise those in [49]: “Tall” = V1-V5, “Medium” = V6-V8, “Short” = V9.
	(iii)	*oviposition site*	“Ground”: eggs laid exclusively in the ground (8 species). “Vegetation”: eggs laid exclusively in vegetation (7 species). “Ground or vegetation”: eggs laid in ground or vegetation (8 species). The latter are species which oviposit at the soil surface or at the base of plants.	“life cycle” sections of species accounts in [21]
	(iv)	*diet*	preferred food of each species: “herbivorous” (14 species) “not herbivorous” i.e. omnivorous or carnivorous (9 species)	species accounts in [21] and [50]
life history	(v)	*mean body size*	mean of minimum and maximum body lengths excluding wings (mean ± s.d. = 16.9 ± 5.9mm); log-transformed	species accounts in [51]
	(vi)	*number of generations per year*	“One”: species requires one year to mature (16 species). “Half”: species always requires at least two years to mature (3 species). “Half or One”: species may develop in one or more years (4 species)	“life cycle” sections of species accounts in [21].
	(vii)	*winter stage*	developmental stage in which the species overwinters: “Egg” (19 species). “Not egg” (i.e. nymph or adult) (4 species)	“life cycle” sections of species accounts in [21]
	(viii)	*phenology*	time of year when adults first appear, to the nearest quarter of a month (mean across species = 6.6, i.e. in the third quarter of June; s.d. = 1.2, i.e. just over one month)	“life cycle” sections of species accounts in [21]
dispersal ability	(ix)	*wing morph*	“Short”: wings never reach to end of abdomen and species is always flightless (3 species). “Long”: wings may reach to end of abdomen or beyond (and species does not display wing-length dimorphism) (13 species). “Dimorphic”: species exhibits wing-length dimorphism (7 species)	species accounts in [21]
	(x)	*wing load*	ratio of the square of a species’ mean wing length (in mm) to the cube of a species’ mean body length (in mm) as calculated in (v) above. Square of wing length was used as proxy for wing area, and cube of body length as proxy for body mass [52], since actual measurements were not available in the literature for all species (mean ± s.d. = 0.051 ± 0.043)	species accounts in [50]. No wing length measurements were available for *Tetrix* species; for these, pronotum lengths in [51] were used instead, which approximate hind wing length [21]. For wing-dimorphic species, wing lengths of macropters were used.
distribution	(xi)	*average latitude*	average latitude of hectads occupied by a species in 1980–9; only “surveyed squares” with at least one species recorded in both 1980–9 and 2000–9 were considered (mean ± s.d. = 51.38 ± 0.46 degrees north)	calculated from Orthoptera Recording Scheme distribution dataset [42, 43]

To avoid potential problems with collinearity between explanatory variables, correlations between traits were investigated using a method employed by [53]: Pearson's correlation tests were calculated between continuous variables, Kendall's correlation tests between categorical variables, and Kruskal-Wallis tests between continuous and categorical variables. A sequential Bonferroni correction was applied in order to account for the large number of tests conducted (55) [54]. No significant correlations were found.

To investigate the relationships between distribution changes and species traits we fitted Generalised Linear Models (GLMs) with Gaussian errors, using first “uncorrected range change” as dependent variable and then repeating analyses with “corrected range change” values. In order to understand the relative importance of different traits in driving distribution changes we took a multimodel inference approach, fitting all possible combinations of trait variables, selecting a set of top models by Akaike information criterion (AIC), and averaging the coefficients and standard errors of trait variables across these [55, 56]: We fitted GLMs for all 2,047 combinations of the 11 explanatory trait variables and calculated AIC values and differences to the best model with the lowest AIC (ΔAIC). Models with ΔAIC < 4 were selected as the top set for which there was considerable statistical support [55]. The percentages of top models in which each trait occurred were then calculated. In order to measure the relative importance of each trait, AIC values were transformed to “Akaike weights” [55, 57], and using these weights, means of trait coefficients across top models were calculated with the “weighted.mean” function in R. Weighted mean standard errors of coefficients were calculated using the following formula adapted from [55]:

$$SE(b_{all})=\;\overset{n}{\underset{i=1}{\sum }}w_{i}\sqrt{{\left[SE\left(b_{i}\right)\right]}^{2}+{\left[b_{i}-b_{all}\right]}^{2}}$$

where n is the number of models, w_i_ is the Akaike weight of model i, SE(b_i_) is the standard error of coefficient b in model i, and b_all_ is the weighted mean of all coefficients b. Akaike weights were scaled so that their sum equalled 1 for each predictive variable, i.e. w_i_ values were divided by the sum of Akaike weights of all models which included the variable whose mean standard error was to be calculated. Confidence intervals (CI) across top models were then calculated by multiplying the weighted mean standard errors with factors of 1.96 (95% CI), 2.58 (99% CI) and 3.29 (99.9% CI) and adding / subtracting them from the weighted means of coefficients. Significance levels were assigned accordingly where the values did not span zero (* for 95% CI, ** for 99% CI, *** for 99.9% CI). Throughout this part of the analysis, range change values calculated from the largest set of “surveyed squares” (with a minimum of one species recorded in both time periods, i.e. with the minimum adequate level of recording effort) were used as our primary measures, and results were then compared to those obtained with the other three sets of “surveyed squares” i.e. higher levels of recording effort, in order to assess the robustness of our findings.

All analyses of relationships between distribution changes and species traits were also repeated with the exclusion of two species with particularly large range change values, *Conocephalus discolor* and *Metrioptera roeselii* (see below).

To assess the validity of using Gaussian GLMs with our data we plotted normal quantile-quantile plots of residuals of top sets of models and carried out Shapiro-Wilk tests for normality [58, 59].

To assess the overall goodness-of-fit of top models the amount of deviance accounted for by each model was calculated:

$$D^{2}=\;\left[null\;deviance\;-\;residual\;deviance\right]\;/\;null\;deviance$$

This was adjusted to take into account the number of observations, i.e. species (s) and the number of predictors, i.e. traits (t) [60, 61]:

$$adjusted\;D^{2}=\;1-\left[\left(s-1\right)/\left(s-t\right)\right]*[1-D^{2}]$$

To give an overall fit of the top models, adjusted D^2^ values were averaged, weighted by AIC weights as with the model coefficients before.

Fitted values of range change were extracted for the top models, and means weighted by model Akaike weights were calculated.

We investigated the potential influence of phylogenetic autocorrelation, i.e. non-independence of trait values due to relatedness between species, based on a method employed by [30]. A “working phylogeny” [62] of the study species was drawn based on the taxonomy of the Orthoptera Species File [63] in the programme “Treemaker” [64] with all branch segment lengths assumed to be equal (S1 Fig). A phylogeny may be approximated in this way based on taxonomic divisions where the true phylogeny is not (fully) known; assuming equal branch lengths and allowing more than two daughters per node reflects the lack of comprehensive detailed knowledge about the order of splitting [62]. The “working phylogeny” was exported in “nexus” format and imported into R. The expected covariance between species was calculated using the “vcv” function in the R package “ape” and Moran’s I autocorrelation indices were calculated on the residuals of each of the top models using the “Moran.I” function. Moran’s I can take values from −1 (perfect negative autocorrelation) to +1 (perfect positive autocorrelation), with values around zero indicating independence of residuals between related species [65–67]. Where Moran’s I indices were significant or near-significant, phylogenetically corrected models were fitted using the “pgls” function in the R package “caper” [68, 69]; as with GLMs before, models were initially fitted to all possible combinations of trait variables and results were then averaged across a set of top models with ΔAIC<4.

## Results

### Range changes

**Fig 2:**
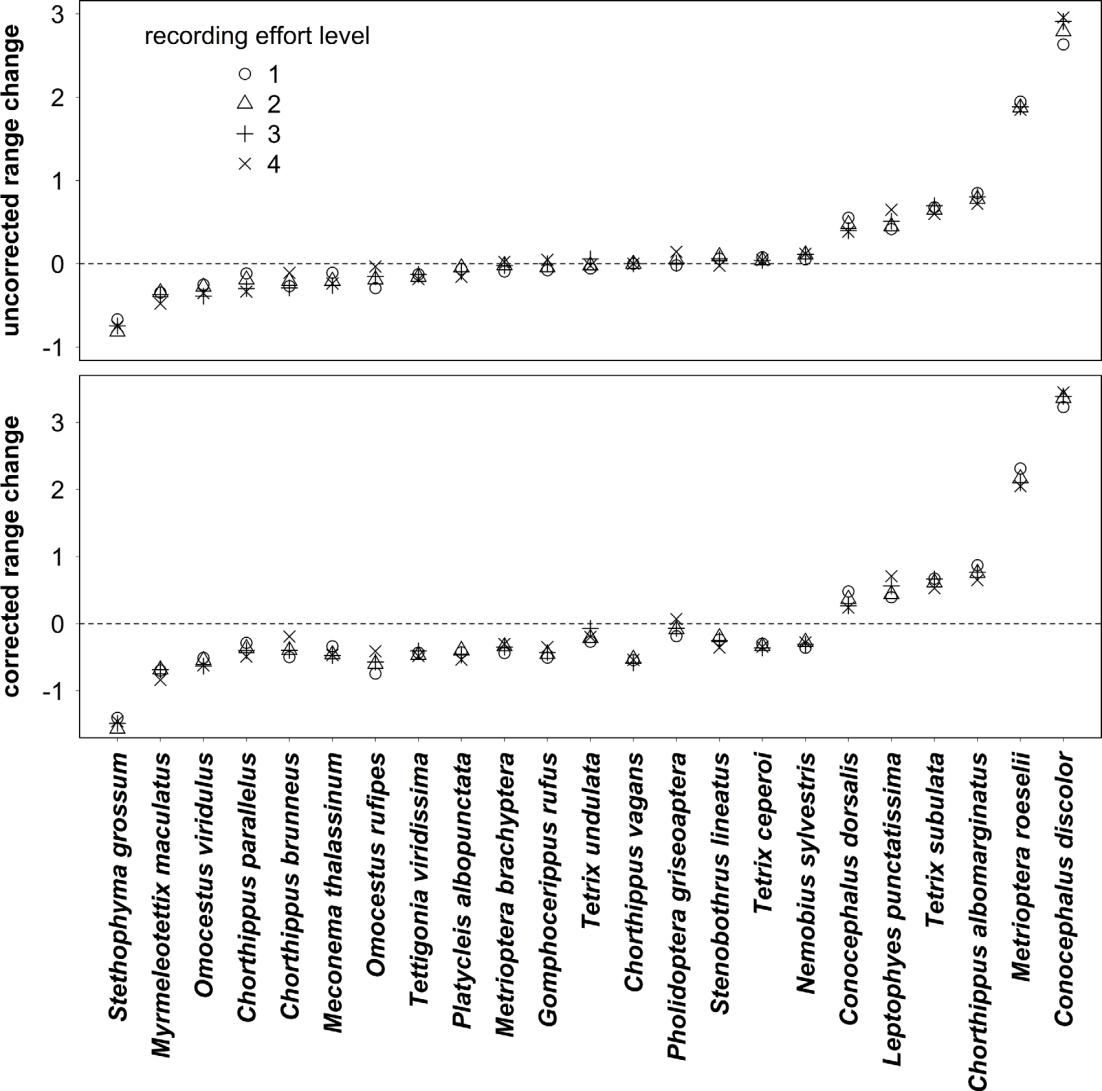
Range changes of grasshoppers and related species in Britain between 1980–9 and 2000–9. The figure shows “uncorrected” and “corrected range change” values for four levels of recording effort—i.e. based on four sets of “surveyed squares” with a minimum of 1 to 4 grasshopper or related species recorded in both time periods. Species are arranged in order of average uncorrected change. Note different y-axis scales.

Our analysis of grasshopper and related insect range changes in Britain between the 1980s and 2000s showed moderate or large range size increases for a few species, with range size decreases for a smaller number, and less or no consistent change for the remaining majority of species. The species with the largest positive range changes were *Conocephalus discolor*, *Metrioptera roeselii*, *Chorthippus albomarginatus* and *Tetrix subulata*; those with the largest range size decreases were *Stethophyma grossum* and *Myrmeleotettix maculatus* (Fig 2).

**Fig 3:**
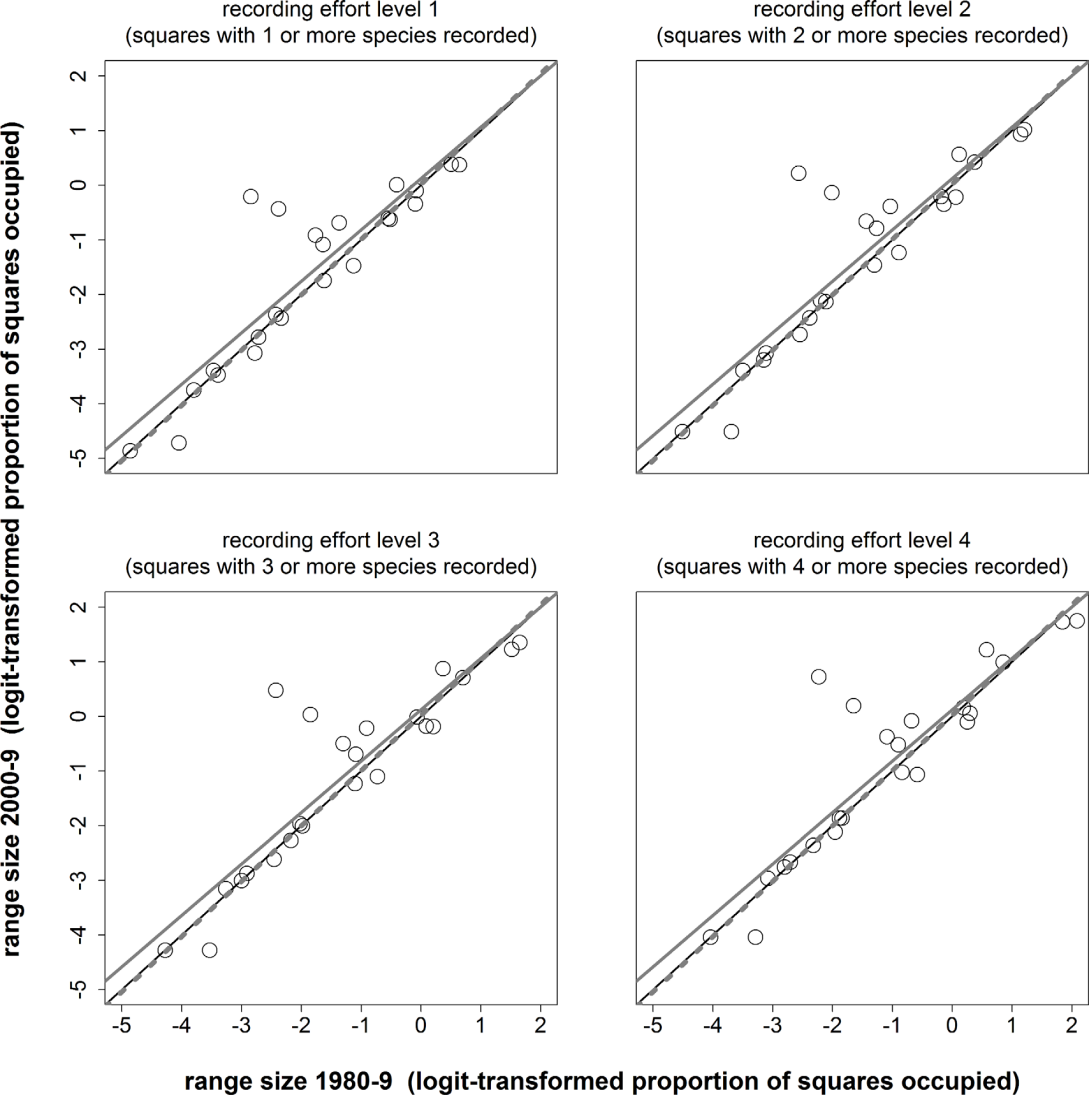
Grasshopper and related species range sizes in 1980–9 and 2000–9 and calculation of range change measures. The figure plots range sizes in 1980–9 vs. 2000–9 (as logit-transformed proportions of squares occupied) for four levels of recording effort. “Uncorrected range change” was defined as the absolute change in range size, i.e. residual distances from the (black) 1:1 unity lines. “Corrected range change” was defined as change in range size relative to the mean change across species, i.e. as the (standardised) residual distances from the linear regression lines (solid grey for all species, dashed grey for species excluding the two with particularly large range change values, *C*. *discolor* and *M*. *roeselii*).

There was a very high degree of consistency of range change values both across levels of recording effort and between “uncorrected” and “corrected” range change measures (Pearson’s r = 0.975 or greater, across all sets of range change values; Fig 3, Table 3, S1 Table).

**Table 3:** Correlation between range change values.

			“uncorrected range change”			“corrected range change”			
			level of recording effort (minimum number of species recorded in “surveyed squares”)			level of recording effort (minimum number of species recorded in “surveyed squares”)			
			2	3	4	1	2	3	4
“uncorrected range change”	level of recording effort (minimum number of species recorded in “surveyed squares”)	1	0.996	0.992	0.983	0.994	0.989	0.985	0.975
		2		0.998	0.993	0.99	0.994	0.991	0.986
		3			0.995	0.983	0.988	0.989	0.984
		4				0.975	0.985	0.986	0.991
“corrected range change”	level of recording effort (minimum number of species recorded in “surveyed squares”)	1					0.996	0.993	0.982
		2						0.998	0.992
		3							0.995

Pearson’s correlation test values between “uncorrected” and “corrected range change” and four levels of recording effort.

Plots of annual relative numbers of hectads recorded per species showed trajectories consistent with our calculated range change values, and none of these annual series were indicative of a one-off population outbreak (S2 and S3 Figs).

Two species, *Conocephalus discolor* and *Metrioptera roeselii* had undergone particularly large range changes compared to the other species (Fig 2). In terms of the observed values of range change, they were statistical outliers (Grubbs’ test for outliers: *C*. *discolor* (G = 3.25, p = 0.0018) and *M*. *roeselii* (G = 3.43, p = 0.0004) for “uncorrected range change”, recording effort level 1). There were equivalent test results for all levels of recording effort and both range change measures (S2 Table). As a matter of caution, therefore, the subsequent traits analysis was repeated with the exclusion of *C*. *discolor* and *M*. *roeselii*, and results compared to those for all species. As detailed below, however, on the basis of the residuals of the trait-based models these species were not statistical outliers so we present and discuss both sets of results.

### Species traits, results for all species

The analysis of relationships between distribution changes and species traits for *all* species showed three traits to be significantly associated with changes in range for both range change measures (Table 4). Firstly, habitat breadth: species that used a greater number of habitats had increased their ranges to a significantly greater extent than those which occurred in fewer habitats (or vice versa) (slope b = 1.38 for uncorrected range change, b = 1.95 for corrected range change, p<0.01 for both). This trait was included in 100% of top models with ΔAIC<4 (top models comprised a set of 47 models for uncorrected, and 53 models for corrected range change). Secondly, oviposition site: for species that oviposited in vegetation, range size increased significantly more than for species that oviposited either in the ground or in the ground or vegetation (or vice versa) (b = 1.07 / b = 0.98 for uncorrected, and b = 1.47 / b = 1.35 for corrected range change, p<0.01 / p<0.05 for both). The oviposition site trait was also included in 100% of top models. The third significant association showed that for species occurring at greater average latitude (i.e. species whose distributions extended further northwards) range size decreased to a greater extent than for species with more southern average latitudes (or conversely, for species with more southern average latitudes range sizes had increased significantly more) (b = -0.76 for uncorrected, b = -0.90 for corrected range change, p<0.05 for both). This trait was included in 100% and 98% of top models for uncorrected and corrected range change respectively.

**Table 4:** Impacts of species traits on distribution changes of British grasshoppers and crickets (all species) between the 1980s and 2000s.

		“uncorrected range change”				“corrected range change”			
	trait	% included	weighted mean coefficient	weighted mean standard error	significance	% included	weighted mean coefficient	weighted mean standard error	significance
(Intercept)		100	39.09	16.36	*	100	45.17	22.48	*
habitat and resource use	(i) breadth of habitat use	100	1.38	0.48	**	100	1.95	0.64	**
	(ii) vegetation structure:	45				45			
	short vs. medium		-0.25	0.38	n.s.		-0.35	0.53	n.s
	short vs. tall		0.49	0.55	n.s.		0.56	0.73	n.s
	medium vs. tall		0.74	0.44	n.s.		0.91	0.57	n.s
	(iii) oviposition site:	100				100			
	vegetation vs. ground		1.07	0.38	**		1.47	0.51	**
	vegetation vs. ground or vegetation		0.98	0.42	*		1.35	0.58	*
	ground vs. ground or vegetation		-0.09	0.29	n.s.		-0.12	0.39	n.s
	(iv) diet:	21				26			
	herbivorous vs. not herbivorous		-0.08	0.35	n.s.		-0.18	0.47	n.s
life history	(v) mean body size	26	-0.50	1.29	n.s.	26	-0.26	1.78	n.s
	(vi) generations per year:	11				9			
	one vs. half		-0.32	0.48	n.s.		-0.45	0.64	n.s
	one vs. half or one		-0.21	0.67	n.s.		-0.25	0.88	n.s
	half vs. half or one		0.12	0.62	n.s.		0.20	0.82	n.s
	(vii) winter stage:	28				30			
	egg vs. not egg		0.01	0.63	n.s.		0.13	0.91	n.s
	(viii) phenology	30	-0.17	0.21	n.s.	38	-0.26	0.29	n.s
dispersal ability	(ix) wing morph:	21				19			
	short vs. long		0.12	0.78	n.s.		0.22	1.06	n.s
	short vs. dimorphic		-0.14	0.92	n.s.		-0.18	1.19	n.s
	long vs. dimorphic		-0.27	0.42	n.s.		-0.40	0.53	n.s
	(x) wing load	40	0.32	0.32	n.s.	38	0.39	0.46	n.s
distribution	(xi) average latitude	100	-0.76	0.32	*	98	-0.90	0.43	*

Summary of results for sets of top GLM models with ΔAIC<4 (47 models for “uncorrected range change”, and 53 models for “corrected range change”). The importance of traits is indicated by the frequency with which they are included in the top model set (% included), and by their weighted mean coefficients, standard errors and significance levels. Significance levels:

* = p<0.05,

** = p<0.01.

Results given are for minimum adequate recording effort, i.e. for “surveyed squares” with a minimum of 1 species recorded in both 1980–9 and 2000–9.

Results were highly consistent across all levels of recording effort and both range change measures, with the same three significant associations found in each case, and significant traits included in similar percentages of top models. Since there were no differences between results with different levels of recording effort, only those for minimum adequate levels of recording effort are presented.

Normal quantile-quantile plots and Shapiro-Wilk tests of residuals of the top sets of models revealed very little deviation from normality: tests had a median p-value of 0.410 for “uncorrected range change”, and a median p-value of 0.418 for “corrected range change”, with between 98% and 100% of top models showing no significant deviation from normality (p> = 0.05) across all levels of recording effort and both range change measures (S3 Table). We therefore concluded that using Gaussian GLMs with our data was valid in this respect.

Similarly, the analysis of phylogenetic autocorrelation in the top GLMs by calculation of Moran’s I indices yielded low, non-significant index values for between 95% and 100% of top models for both range change measures and all levels of recording effort (S4 Table). Subsequent fitting and selection of phylogenetically corrected “pgls” models did not change the results obtained with non-phylogenetic GLMs: The same numbers of top models were selected, with the same predictors and virtually identical coefficient and p-values as in GLMs (S5 Table). Lambda values were consistently estimated as the default minimum permitted in the pgls function, 1x10^-6^. We therefore concluded that these results were indicative of a low phylogenetic signal and hence the analysis with non-phylogenetically-corrected GLMs was robust.

Calculation of adjusted D^2^ values showed fairly high overall goodness-of-fit across top models with *all* species: the weighted means of adjusted D^2^ values were 0.54 (minimum 0.03, maximum 0.59) and 0.56 (minimum 0.03, maximum 0.61) for “uncorrected” and “corrected range change” respectively. These were the values for the minimum adequate level of recording effort, and very similar ones were obtained for higher levels of recording effort (S6 Table).

### Species traits, results excluding Conocephalus discolor and Metrioptera roeselii

When the analysis of the relationships between distribution changes and species traits by GLMs was repeated for all species *excluding* the two species with particularly large range changes, *Conocephalus discolor* and *Metrioptera roeselii*, no traits were found to be significantly associated with changes in range for either measure of range change (Table 5).

**Table 5:** Impacts of species traits on distribution changes of British grasshoppers and crickets (excluding *Conocephalus discolor* and *Metrioptera roeselii*) between the 1980s and 2000s.

		“uncorrected range change”				“corrected range change”			
	trait	% included	weighted mean coefficient	weighted mean standard error	significance	% included	weighted mean coefficient	weighted mean standard error	significance
(Intercept)		100	0.98	3.12	n.s.	100	3.71	8.94	n.s.
habitat and resource use	(i) breadth of habitat use	49	0.38	0.30	n.s.	47	0.97	0.88	n.s.
	(ii) vegetation structure:	3				5			
	short vs. medium		-0.13	0.24	n.s.		-0.31	0.73	n.s.
	short vs. tall		-0.01	0.30	n.s.		0.16	0.93	n.s.
	medium vs. tall		0.12	0.25	n.s.		0.46	0.71	n.s.
	(iii) oviposition site:	24				22			
	vegetation vs. ground		0.24	0.24	n.s.		0.56	0.73	n.s.
	vegetation vs. ground or vegetation		0.04	0.26	n.s.		-0.01	0.79	n.s.
	ground vs. ground or vegetation		-0.20	0.18	n.s.		-0.57	0.51	n.s.
	(iv) diet:	20				18			
	herbivorous vs. not herbivorous		-0.03	0.19	n.s.		-0.06	0.53	n.s.
life history	(v) mean body size	59	-1.06	0.72	n.s.	67	-3.24	2.08	n.s.
	(vi) generations per year:	2				4			
	one vs. half		0.02	0.24	n.s.		0.17	0.70	n.s.
	one vs. half or one		0.04	0.27	n.s.		0.29	0.80	n.s.
	half vs. half or one		0.02	0.34	n.s.		0.12	0.94	n.s.
	(vii) winter stage:	32				33			
	egg vs. not egg		-0.21	0.36	n.s.		-0.68	1.04	n.s.
	(viii) phenology	38	0.12	0.11	n.s.	42	0.38	0.31	n.s.
dispersal ability	(ix) wing morph:	15				8			
	short vs. long		0.18	0.28	n.s.		0.43	0.74	n.s.
	short vs. dimorphic		-0.07	0.31	n.s.		-0.17	0.77	n.s.
	long vs. dimorphic		-0.25	0.20	n.s.		-0.60	0.58	n.s.
	(x) wing load	18	-0.03	0.17	n.s.	18	-0.05	0.45	n.s.
distribution	(xi) average latitude	25	-0.05	0.23	n.s.	24	-0.21	0.64	n.s.

Summary of results for sets of top GLM models with ΔAIC<4 (95 models for “uncorrected range change”, and 79 models for “corrected range change”). The importance of traits is indicated by the frequency with which they are included in the top model set (% included), and by their weighted mean coefficients, standard errors and significance levels. Results given are for minimum adequate recording effort, i.e. for “surveyed squares” with a minimum of 1 species recorded in both 1980–9 and 2000–9.

As before in the analysis with all species, results were highly consistent across all levels of recording effort and both range change measures, therefore only the results for minimum adequate levels of recording effort are presented. Residuals were normally distributed indicating that the analysis with Gaussian GLMs was robust (S3 Table). There were significant results for Moran’s I phylogenetic autocorrelation indices for up to about half of the top models, but index values were low throughout (S4 Table). In addition, subsequent fitting of phylogenetically corrected pgls models did not change the results obtained with non-phylogenetic GLMs (S7 Table), and lambda values were consistently estimated as the default minimum permitted in the pgls function, 1x10^-6^. We therefore concluded that the analysis with non-phylogenetically-corrected GLMs was robust.

Goodness-of-fit was drastically reduced for models that *excluded* the two species with particularly large range changes compared to models with all species: the weighted means of adjusted D^2^ values were 0.12 (minimum 0.00, maximum 0.24) for both range change measures and were very similar across all levels of recording effort (S6 Table).

**Fig 4:**
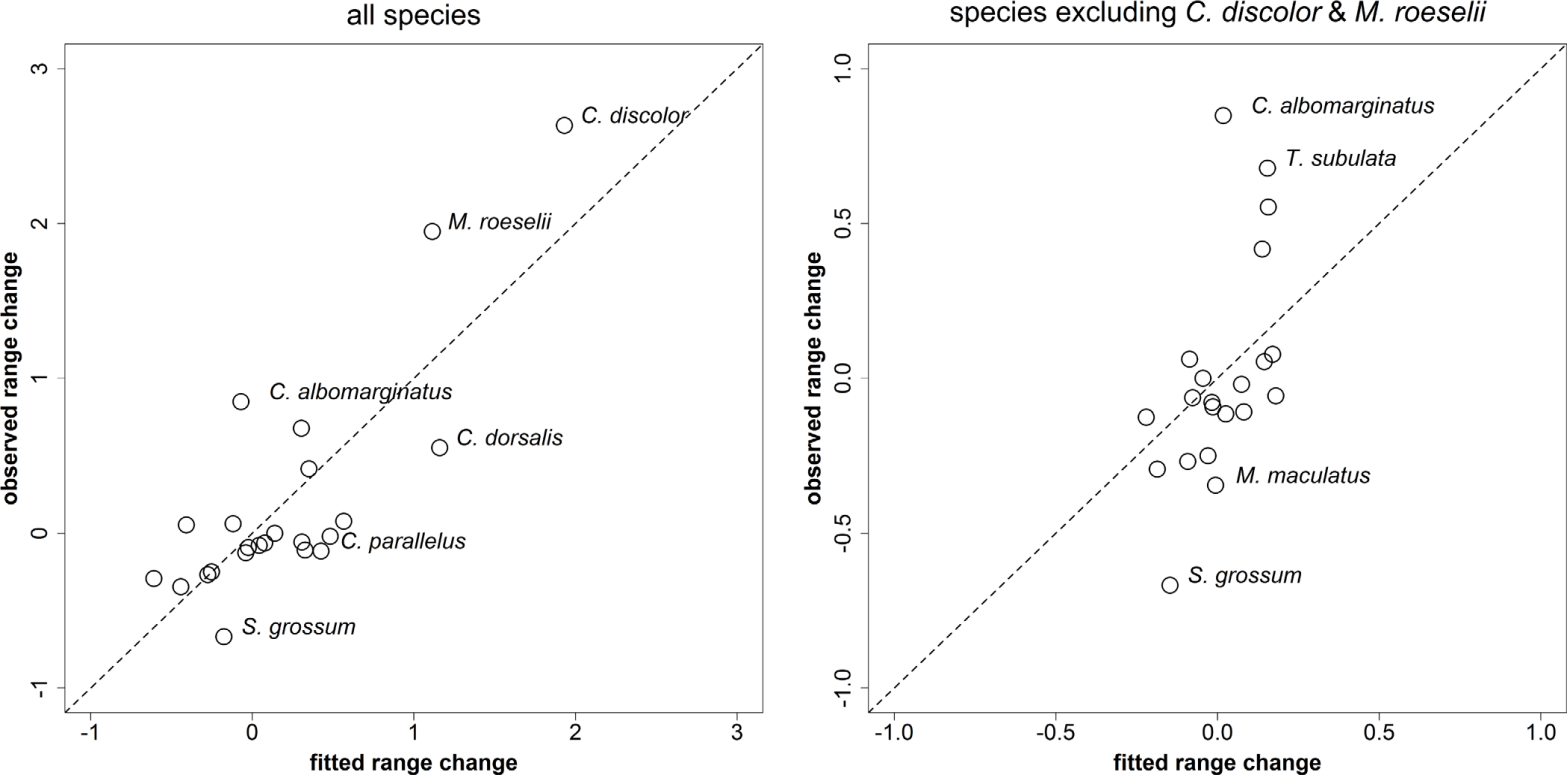
Observed vs. fitted range change values. Values for “uncorrected range change”, recording effort level 1. Fitted values are weighted means across the set of top GLM models with ΔAIC<4. The dashed unity line indicates equality of observed and fitted values. Species with the largest residuals have been labelled.

For models with all species, despite a good average correspondence between observed and fitted range change values, there were large residuals for some species (Fig 4). For models including all species, the species that had the largest positive differences between observed and fitted values (i.e. most underestimated by the models) were *Chorthippus albomarginatus*, *M*. *roeselii* and *C*. *discolor*; those with the largest negative differences (i.e. range changes most overestimated by the models) were *Conocephalus dorsalis*, *Chorthippus parallelus* and *Stethophyma grossum*. For models excluding *C*. *discolor* and *M*. *roeselii* the species with the largest positive differences were again *C*. *albomarginatus* and also *Tetrix subulata*, those with the largest negative differences were again *S*. *grossum* and also *Myrmeleotettix maculatus*. Results were very similar across measures of range change and levels of recording effort (S8 Table).

## Discussion

Considerable distributional changes have occurred among British grasshoppers and related species in recent decades. Our analysis provides interesting indications as to which combination of traits is responsible for the particularly large range expansions of two species, *C*. *discolor* and *M*. *roeselii*. No effects were found and model fits dropped sharply when as a matter of caution these two species were omitted, and conclusions about the importance of specific traits therefore had limited relevance to the remaining species. Limited predictive and explanatory power is a common feature of traits analyses in the literature—while a number of studies find significant associations, the variation explained is generally low, and the traits that are identified for a taxonomic group may vary between studies [8, 9, 70–72]. It is likely that characteristics of species beyond those examined explain additional variation, e.g. physiology, trophic relationships, or interactions between traits, but this remains to be demonstrated and will require more information than is currently available. For example, there is limited data on physiological tolerances and quantitative importance of food-web interactions for grasshoppers and relatives [21, 73]. Additional constraints of our study were the small number of species (23), which meant that for traits with few species in individual categories there was limited statistical power, and the necessity to employ conservative range change measures which, while robust, are unable to detect small distributional changes, or indeed more subtle changes in abundance. In the discussion of the findings of the traits analysis we restrict application mainly to *C*. *discolor* and *M*. *roeselii* because they have a dominant effect on results.

### Range changes

Both our measures of range change, “uncorrected” and “corrected”, control for spatial variation in recording and the latter measure is also robust to overall changes in recording effort and multiple other biases [47]. Given this and the very close correlation of values across both range change measures and all levels of recording effort (Table 3), we are confident that they are robust, if conservative, estimates of range change.

**Fig 5:**
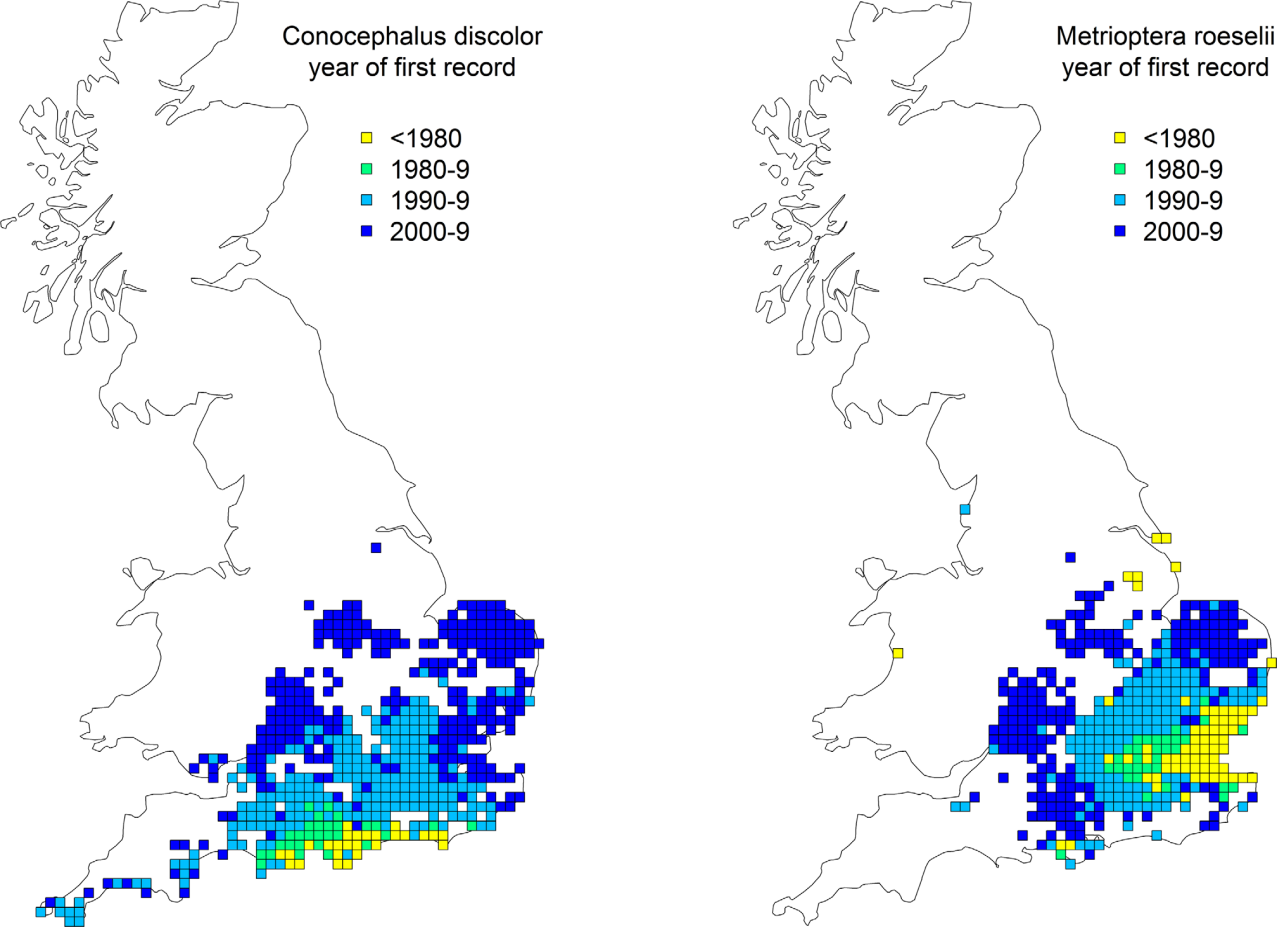
Range expansions of *Conocephalus discolor* and *Metrioptera roeselii* in Britain between 1980 and 2009. The figure shows years of first records of the species in each hectad. N.B.: The maps are based on the dataset retrieved from the Orthoptera Recording Scheme database for the present study (in 2013).

In Britain, those grasshoppers and crickets which have restricted ranges are generally confined to the south or south-east, i.e. they are limited to the warmer and drier regions and have a range margin towards the north or north-west, presumably due to physiological constraints [21]. Consequently, where range expansions occurred, they proceeded in predominantly northerly and westerly directions. For example, this can be clearly seen in the two species with the greatest range increases in this study, *C*. *discolor* and *M*. *roeselii* (Fig 5). Such north- or northwest-ward range expansions are also consistent with a climatic explanation (see discussion of average latitudes below).

Populations of grasshoppers and crickets may undergo large fluctuations in density from year to year, for example in response to variations in abiotic factors such as temperature and precipitation, with densities varying by factors of up to 5 or 10 or even more between successive years [73]. These fluctuations in density may in turn lead to fluctuations in distributions, particularly at small scales [74]. If fine-scale records of individual years were to be compared, therefore, erroneous conclusions might be reached about changing distributions. Here, we summarised records at a coarse spatial scale (10x10km squares), and examined distribution changes across whole decades (1980s vs. 2000s) [5, 46]. We are confident, therefore, that any substantial range changes observed reflect genuine change. Comparison of trajectories of change between decades with those inferred from annual series of records over the entire study period 1980–2009 confirm that large observed range changes are genuine, cumulative, and sustained and are not artefacts of one-off fluctuations or outbreaks (S2 and S3 Figs).

### Species traits

Our all-species traits analysis found three species traits to have significant effects on range changes between the 1980s and 2000s (Table 4). The observed significant positive effect of the number of habitats that a species utilises on its ability to extend its distribution has been documented in several species groups and is consistent with the notion that under conditions of environmental change species with a broad ecological niche are more likely to be able to find suitable resources in the landscape than specialists [6, 23, 29]. The species with the largest range size increases in our study, the bush-crickets *C*. *discolor* and *M*. *roeselii* are both habitat generalists occurring in many long-grass habitats. Both are likely to have benefited from “set-aside”, i.e. the large areas of agricultural land left untilled in the 1990s and 2000s under farming policy, and field margins taken out of production under the subsequent “agri-environment schemes”; in addition they occur along lightly managed roadsides, railway lines and flood defences, whose linear nature may have further enhanced connectivity of suitable habitats [19, 21]. Potential links between the number of habitats species can exploit and climate warming are discussed below.

The second finding of our all-species traits analysis—a significant effect of oviposition site, with species which lay their eggs in vegetation increasing their ranges more than species that oviposit in the ground or at the ground-vegetation interface—may be related to land use changes and their effects on microclimates. Britain’s large-scale “Countryside Survey 2007” found many indications of reduced management, and nutrient enrichment in some habitats, both in the short (since 1998/1990) and longer term (since 1978), with vegetation becoming taller, more shaded and less diverse [16]. The recently published second atlas of mosses and liverworts in Britain documents particular declines for species of low-nutrient lowland habitats [75]. Notwithstanding localised decreases in vegetation height through factors such as increasing rabbit populations [76] and targeted habitat management, therefore, it is possible that suitable microclimates for insects that oviposit in the ground have generally decreased, despite climatic warming. At the same time, species that oviposit in vegetation including the two with the largest range size increases in the present study, *M*. *roeselii* and *C*. *discolor*, may have benefited from climatic warming without suffering negative effects from increases in vegetation height. Conversely, the mottled grasshopper *Myrmeleotettix maculatus* has shown one of the largest declines in our study; it oviposits in the soil and is a specialist of short vegetation and bare ground exposed to the sun and is likely to be very vulnerable to succession and nutrient enrichment [21, 50]. The importance of short vegetation or open ground for oviposition have been highlighted for other taxa such as bumblebees [77], butterflies [35], moths [7] and indeed recently for grasshoppers and relatives with an explicit link to a negative effect of nutrient enrichment [26].

The third finding of our all-species traits analysis was a significant positive effect of low (southerly) average latitude of a species’ distribution on range size. This is consistent with a positive effect of climatic warming over the study period 1980–2009: Being on their northern range edge, species with low average British latitudes such as *M*. *roeselii* and *C*. *discolor* are likely to be thermally constrained, i.e. their distributions limited by their minimum physiological requirements for warmth. Under a warming climate they are therefore expected to expand their ranges into previously unsuitable areas; such changes have been observed for multiple species groups [2, 5, 78]. Consistent with this explanation, *M*. *roeselii* and *C*. *discolor* have also been extending their ranges in continental Europe [79–81]. There is a possibility that due to the concentration of “surveyed squares” in southern Britain (Fig 1) it is easier to detect change in the more thermally limited species that occur at low average latitudes. However, it is unlikely that range changes of the magnitude observed here (in excess of 300%) would be missed even in regions with low recording intensity. In addition, expanding species would be expected to increase their distributions even away from the immediate range margin through “infill” ([82], and see next paragraph).

An interesting aspect of species’ responses to climatic warming is the interaction with habitat breadth: populations located near species’ thermal limits are often confined to fewer habitats than elsewhere in their range (presumably to those which provide optimum microclimatic conditions) [83]. Climatic warming should therefore increase the range of habitats available to them (“ecological release”), and instances of this have been documented [84, 85], although other studies have failed to find such an effect, presumably because of concurrent habitat deteriorations due to other factors [86]. There is anecdotal evidence that *C*. *discolor* and *M*. *roeselii* (and the species with the third largest positive range change in our study, *Chorthippus albomarginatus*) have increased the numbers of habitats they utilise in Britain during their recent range expansions [21, 48], but no specific studies have been carried out and the observed changes may be density-dependent or determined by land-use changes rather than climate-driven. Some of Britain’s rarest grasshoppers and relatives are very specialised here but occur in a wider range of habitats away from the edge of their range, in continental Europe, for example the species with the largest range contraction in our study, *Stethophyma grossum* [21, 51]. It may be that continued climatic warming will aid conservation of such species in Britain by allowing them to occupy additional habitats, but this will depend on other conditions such as moisture levels also meeting the species’ requirements [21, 87, 88].

Another interesting mechanism by which climatic warming could aid range expansions is through increases in voltinism [37]. The development of *M*. *roeselii* (and that of a second species which has expanded its range, *Leptophyes punctatissima*) can take either one or two years (Table 1): eggs laid early in the season and/or in warm parts of the species’ range take one year to develop into adults, while eggs laid late or in cooler parts of the range overwinter twice before hatching [89, 90]. Increased temperatures could therefore halve generation times for parts of the populations of these species and so aid increases in numbers and range expansions. The number of generations per year is not identified as a significant trait in our analysis. This may be because the trait is too coarse to capture inter- and intra- specific *variability* in voltinism adequately: For example, *Tettigonia viridissima* (“half” a generation per year) may take two or more years to develop, and females of *Chorthippus brunneus* (“one” generation per year) exhibit seasonal and regional variability in the number of instars during development (four or five), with early and southerly eggs more likely to develop through five instars, producing larger and more fecund adults [17, 21]. A further reason that we found no effect here may be that climatic warming may of course also aid reproduction in species such as *C*. *discolor* where no variation in voltinism is known to occur: warming may extend the breeding season, and increase metabolic rates and hence fecundity of adults [17].

In addition to the three traits discussed above, wing-length dimorphism is known to be a further very significant trait catalysing the rapid range expansion of *M*. *roeselii* and *C*. *discolor*: multiple studies suggest both species are expanding their ranges successfully through a combination of effective dispersal (aided by high numbers of macropterous individuals) and subsequent high reproductive rates (of brachypters); selection for increased dispersal at the advancing range margin appears to be reinforcing the process [18, 19, 40, 80, 91, 92]. Wing-length is not identified as a significant predictor of range change in our analysis. Likely reasons for this include that other wing-dimorphic species have not expanded rapidly, and that our study did not take account of maximum proportions of macropters in populations, because the small total number of species did not allow a finer categorisation. In *M*. *roeselii* and *C*. *discolor* populations, macropters may reach very high proportions, while in most other wing-dimorphic species they are never more than rare [21, 50, 51] and therefore presumably have little impact on rapid dispersal at the population level.

Overall, it seems likely that a *combination* of favourable traits is required for species to have been able to expand their ranges under the climatic and land-use changes of recent decades. Wing-dimorphic species such as *C*. *discolor* and *M*. *roeselii* which combine effective dispersal through large numbers of macropters with a broad ecological niche and oviposition preferences suited to recent land-use change have benefited greatly from climatic warming and expanded their range rapidly. It is instructive to compare these species to others which share some but not all of these traits: For example, *Conocephalus dorsalis* is very similar to *M*. *roeselii* in all three traits identified as significant in our study (Table 1), but has expanded its range much less (Fig 2). This may be because, while wing-dimorphic, it is not known to produce large numbers of macropters [21]. A lack of information on maximum proportions of macropters in our analysis may also explain why the range change for this species is overestimated by models, while it is underestimated for *C*. *discolor* and *M*. *roeselii* (Fig 4). Another species, *Chorthippus parallelus*, has somewhat less in common with *C*. *discolor* and *M*. *roeselii*: it is a habitat generalist, is wing-dimorphic and can produce very large proportions of macropters [50, 51], but it oviposits in the ground and has a higher average distributional latitude (Table 1); the range of this species seems in fact to have declined (Fig 2).

## Conclusions

Long-term distributional datasets are a valuable resource that can inform research on species’ responses to environmental change. Our analysis showed large changes in distributions for some grasshoppers and crickets at the scale of a whole geographical region (Britain) between 1980 and 2009, a period of extensive climatic and land use change. Range changes were positively influenced by three species traits: habitat generalism, oviposition above ground in vegetation, and a southerly distribution. However, these findings applied mainly to the two species with the greatest increases in range only, *C*. *discolor* and *M*. *roeselii*, as no effects were found for a subset of species excluding them. Several previous studies on the rapid range expansion of these two species emphasised wing-length dimorphism as the key to their success, with the ability of populations to develop large proportions of long-winged (macropterous) individuals resulting in a high phenotypic plasticity of dispersal. Our findings suggest that dispersal is not the whole picture and that it is likely to be the combination of traits that these species possess that have enabled them to thrive under recent environmental changes. Differences in their traits, however, were not significant predictors of the range size changes of the remaining individual species. We conclude that trait-based analyses may contribute to the assessment of species responses to environmental change and may provide insights into underlying mechanisms, but results need to be interpreted with caution and may have limited predictive power, particularly where trait and population trend data is not extremely detailed and species numbers are low. Advances in species distribution and abundance monitoring, and assembly of more detailed and comprehensive trait data for example alongside the collection of distribution data [93] or through follow-up investigations on the findings of studies such as the present one, will be important for future improvements in assessing the consequences of environmental change.

## Supporting Information

S1 Fig “Working phylogeny” of grasshoppers and related species in Britain. (PDF)

S2 Fig Scatter- and boxplots of annual relative numbers of hectad records for species with the greatest positive range changes. (PDF)

S3 Fig Scatter- and boxplots of annual relative numbers of hectad records for species with the greatest negative range changes. (PDF)

S1 Table Grasshopper and related species range changes between 1980–9 and 2000–9. Range sizes and “uncorrected” and “corrected” range change values for four levels of recording effort.
(PDF)

S2 Table Results of Grubbs’ tests for outliers. Test statistics (G) and p-values (p) for “uncorrected” and “corrected range change” and four levels of recording effort. In each case, *Conocephalus discolor* and *Metrioptera roeselii* were identified as outliers.
(PDF)

S3 Table Results of Shapiro-Wilk tests for normality of residuals. (PDF)

S4 Table Moran’s I phylogenetic autocorrelation indices and associated p-values. (PDF)

S5 Table Impacts of species traits on distribution changes of British grasshoppers and crickets (all species), phylogenetic models. (PDF)

S6 Table Amount of overall variation explained by models (adjusted deviance D^2^). (PDF)

S7 Table Impacts of species traits on distribution changes of British grasshoppers and crickets (excluding *Conocephalus discolor* and *Metrioptera roeselii*), phylogenetic models. (PDF)

S8 Table Fitted range change values. (PDF)
