# Supplementary material for: Two Species with an Unusual Combination of Traits Dominate Responses of British Grasshoppers and Crickets to Environmental Change
Source: PLoS One. 2015 Jun 25;10(6):e0130488. doi: 10.1371/journal.pone.0130488 (PMC4482502; doi:10.1371/journal.pone.0130488)
Supplement: S1 Fig — (PDF) [file pone.0130488.s001.pdf]

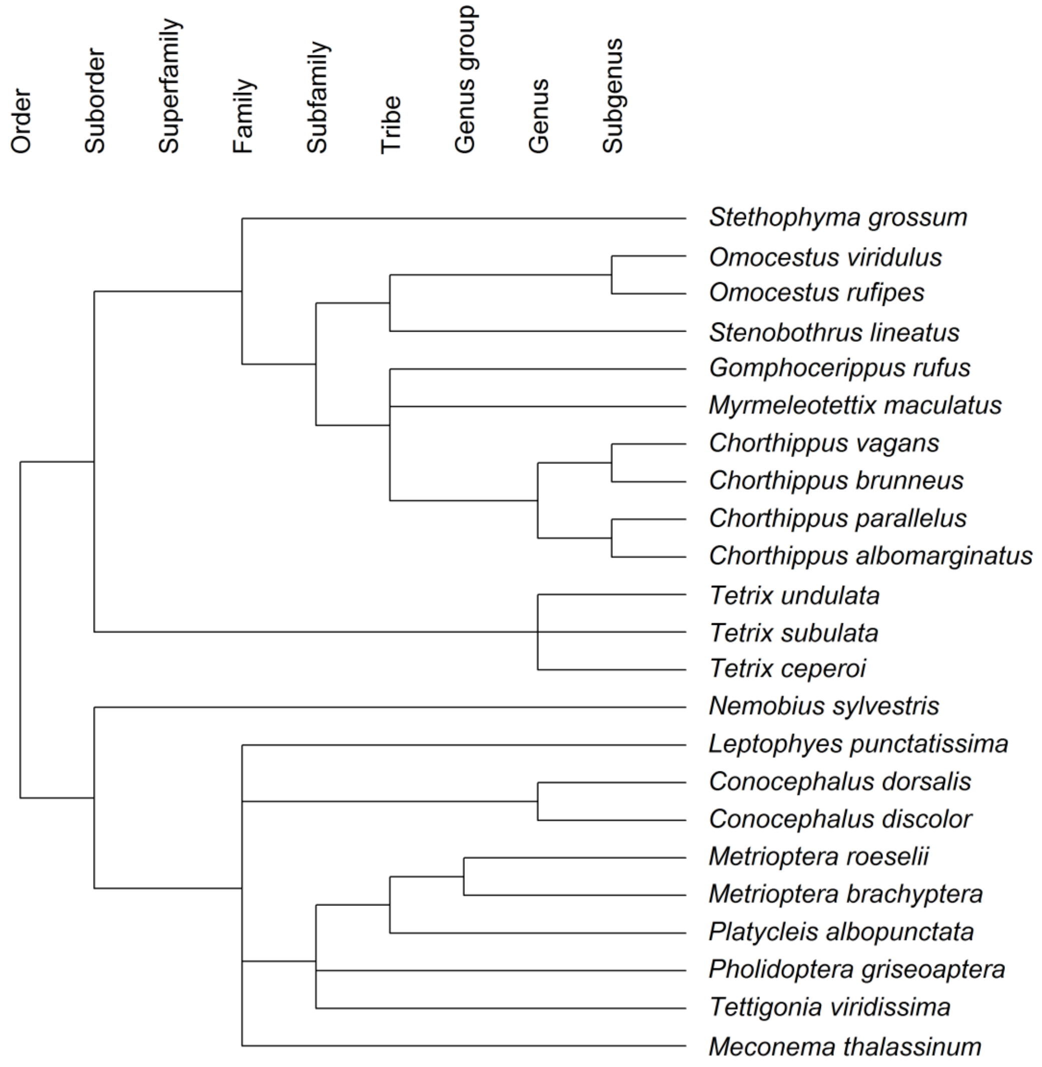

S1 Fig. "Working phylogeny" of grasshoppers and related species in Britain. In the absence of a comprehensive phylogeny of the study species, this "working phylogeny" was approximated based on taxonomic divisions according to the Orthoptera Species File, with all branch segment lengths assumed to be equal [62, 63].

N.B.: In keeping with currently prevailing use in Britain and in line with the rest of the text, some species names have been altered from the Orthoptera Species File, and subgenus names are not included: *Omocestus (Omocestus) viridulus = Omocestus viridulus; Omocestus (Omocestus) rufipes = Omocestus rufipes; Chorthippus (Glyptobothrus) vagans = Chorthippus vagans; Chorthippus (Glyptobothrus) brunneus = Chorthippus brunneus; Chorthippus (Chorthippus) parallelus = Chorthippus parallelus; Chorthippus (Chorthippus) albomarginatus = Chorthippus albomarginatus; Conocephalus (Anisoptera) dorsalis = Conocephalus dorsalis; Conocephalus (Xiphidion) fuscus = Conocephalus discolor; Roeseliana roeselii = Metrioptera roeselii.*
