# Supplementary material for: Two Species with an Unusual Combination of Traits Dominate Responses of British Grasshoppers and Crickets to Environmental Change
Source: PLoS One. 2015 Jun 25;10(6):e0130488. doi: 10.1371/journal.pone.0130488 (PMC4482502; doi:10.1371/journal.pone.0130488)
Supplement: S3 Fig — (PDF) [file pone.0130488.s003.pdf]

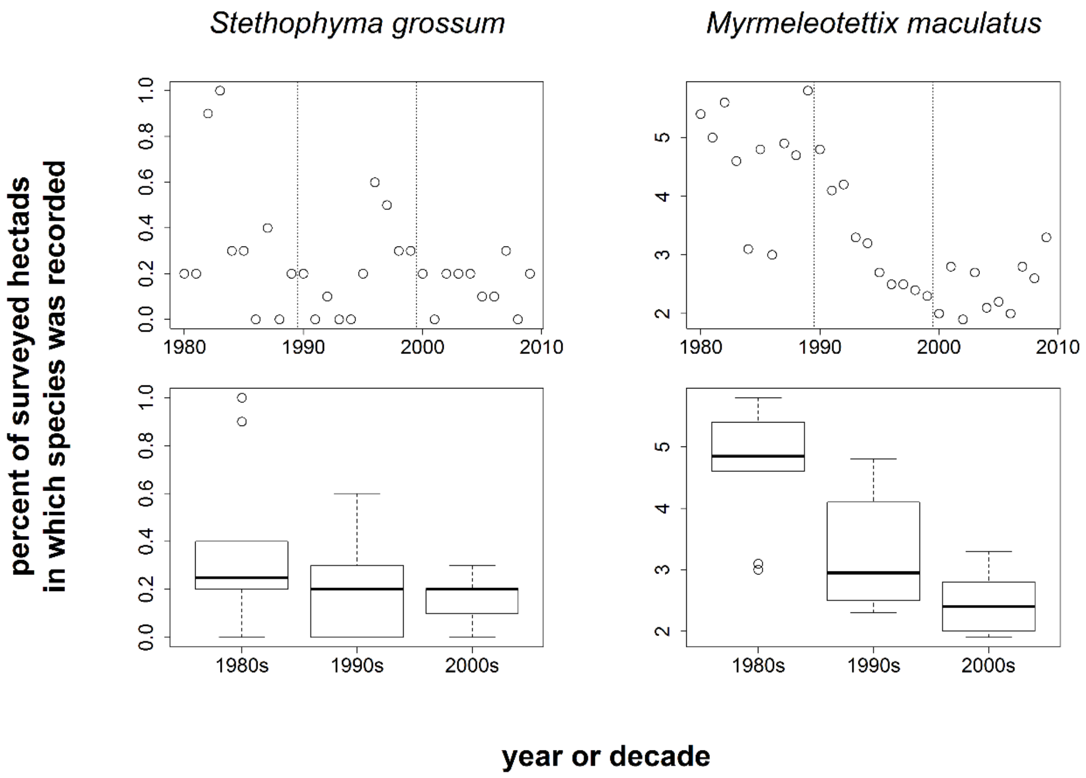

S3 Fig. Scatter- and boxplots of annual relative numbers of hectad records for species with the greatest negative range changes. To account for changes in overall recording effort, annual numbers of hectads in which a species was recorded were calculated as a percentage of the total number of hectads surveyed in the respective year.
