# Supplementary material for: Two Species with an Unusual Combination of Traits Dominate Responses of British Grasshoppers and Crickets to Environmental Change
Source: PLoS One. 2015 Jun 25;10(6):e0130488. doi: 10.1371/journal.pone.0130488 (PMC4482502; doi:10.1371/journal.pone.0130488)
Supplement: S1 Table — Range sizes and “uncorrected” and “corrected” range change values for four levels of recording effort. (PDF) [file pone.0130488.s004.pdf]

S1 Table. Grasshopper and related species range changes between 1980-9 and 2000-9. Range sizes and "uncorrected" and "corrected" range change values for four levels of recording effort.

	based on hectads with >=1 species recorded in both time periods					based on hectads with >=2 species recorded in both time periods					based on hectads with >=3 species recorded in both time periods					based on hectads with >=4 species recorded in both time periods				
species	no. of 10km squares occupied 1980-9	no. of 10km squares occupied 2000-9	"uncorrected range change"	"corrected range change" , all species	"corrected range change" , excluding *C. discolor* and *M. roeselii*	no. of 10km squares occupied 1980-9	no. of 10km squares occupied 2000-9	"uncorrected range change"	"corrected range change" , all species	"corrected range change" , excluding *C. discolor* and *M. roeselii*	no. of 10km squares occupied 1980-9	no. of 10km squares occupied 2000-9	"uncorrected range change"	"corrected range change" , all species	"corrected range change" , excluding *C. discolor* and *M. roeselii*	no. of 10km squares occupied 1980-9	no. of 10km squares occupied 2000-9	"uncorrected range change"	"corrected range change" , all species	"corrected range change" , excluding *C. discolor* and *M. roeselii*
Meconema thalassinum	315	294	-0.11	-0.34	-0.41	277	247	-0.20	-0.46	-0.68	247	216	-0.26	-0.48	-0.72	215	193	-0.24	-0.47	-0.71
Tettigonia viridissima	139	125	-0.13	-0.43	-0.39	127	112	-0.16	-0.47	-0.47	118	107	-0.13	-0.41	-0.34	113	99	-0.18	-0.48	-0.52
Pholidoptera griseoaptera	405	401	-0.02	-0.19	-0.18	353	360	0.05	-0.09	0.02	317	318	0.01	-0.07	0.03	263	274	0.14	0.07	0.34
Platycleis albopunctata	52	49	-0.06	-0.43	-0.16	50	48	-0.04	-0.39	-0.1	48	44	-0.10	-0.47	-0.25	46	40	-0.16	-0.54	-0.41
Metrioptera brachyptera	74	68	-0.09	-0.44	-0.26	64	63	-0.02	-0.34	-0.03	57	56	-0.02	-0.35	-0.05	49	50	0.02	-0.30	0.08
Metrioptera roeselii	71	332	1.95	2.31	-	70	278	1.88	2.17	-	64	241	1.88	2.10	-	60	206	1.85	2.05	-
Conocephalus discolor	46	378	2.63	3.23	-	42	331	2.79	3.36	-	38	293	2.91	3.39	-	36	253	2.96	3.45	-
Conocephalus dorsalis	137	213	0.55	0.48	1.53	131	186	0.48	0.37	1.31	119	158	0.40	0.27	1.09	108	140	0.39	0.24	1.05
Leptophyes punctatissima	337	424	0.42	0.39	1.1	315	380	0.45	0.44	1.2	280	335	0.51	0.56	1.43	240	290	0.65	0.71	1.77
Nemobius sylvestris	18	19	0.05	-0.36	0.24	17	19	0.11	-0.27	0.42	17	19	0.11	-0.31	0.33	16	18	0.12	-0.28	0.4
Tetrix ceperoi	25	27	0.08	-0.30	0.29	25	26	0.04	-0.34	0.19	24	25	0.04	-0.36	0.13	23	24	0.04	-0.34	0.17
Tetrix subulata	171	282	0.68	0.67	1.87	156	241	0.65	0.61	1.79	136	212	0.70	0.67	1.9	126	180	0.60	0.53	1.63
Tetrix undulata	309	298	-0.06	-0.27	-0.26	270	267	-0.02	-0.21	-0.15	229	236	0.06	-0.07	0.17	205	203	-0.02	-0.20	-0.1
Stethophyma grossum	14	7	-0.67	-1.40	-1.9	14	6	-0.82	-1.57	-2.32	13	6	-0.75	-1.49	-2.12	13	6	-0.75	-1.46	-2.1
Stenobothrus lineatus	68	72	0.06	-0.24	0.18	59	64	0.09	-0.20	0.28	55	58	0.06	-0.25	0.17	51	50	-0.02	-0.36	-0.05
Omocestus rufipes	49	37	-0.29	-0.74	-0.81	43	36	-0.19	-0.60	-0.5	37	32	-0.15	-0.57	-0.42	33	32	-0.03	-0.41	-0.06
Omocestus viridulus	402	350	-0.25	-0.51	-0.85	307	266	-0.28	-0.54	-0.9	261	215	-0.39	-0.63	-1.07	211	178	-0.35	-0.62	-1.03
Chorthippus brunneus	553	500	-0.27	-0.50	-0.98	452	428	-0.21	-0.40	-0.8	389	367	-0.29	-0.40	-0.84	324	319	-0.11	-0.19	-0.42
Chorthippus vagans	6	6	0.00	-0.54	0.14	6	6	0.00	-0.52	0.16	6	6	0.00	-0.58	0.01	6	6	0.00	-0.54	0.09
Chorthippus parallelus	526	503	-0.11	-0.29	-0.5	459	438	-0.19	-0.37	-0.75	398	377	-0.30	-0.40	-0.88	334	320	-0.33	-0.49	-1.12
Chorthippus albomarginatus	123	241	0.85	0.87	2.37	114	203	0.78	0.76	2.18	101	179	0.80	0.77	2.19	94	153	0.72	0.65	1.98
Gomphocerippus rufus	27	25	-0.08	-0.51	-0.17	24	23	-0.04	-0.45	-0.05	22	22	0.00	-0.43	0.01	21	22	0.05	-0.35	0.18
Myrmeleotettix maculatus	206	157	-0.34	-0.70	-1.04	173	134	-0.34	-0.69	-1.02	154	118	-0.37	-0.69	-1.01	134	96	-0.48	-0.84	-1.34
