# Supplementary material for: Two Species with an Unusual Combination of Traits Dominate Responses of British Grasshoppers and Crickets to Environmental Change
Source: PLoS One. 2015 Jun 25;10(6):e0130488. doi: 10.1371/journal.pone.0130488 (PMC4482502; doi:10.1371/journal.pone.0130488)
Supplement: S2 Table — Test statistics (G) and p-values (p) for “uncorrected” and “corrected range change” and four levels of recording effort. In each case, Conocephalus discolor and Metrioptera roeselii were identified as outliers. (PDF) [file pone.0130488.s005.pdf]

S2 Table. Results of Grubbs' tests for outliers.

		"uncorrected range change"				"corrected range change"			
		level of recording effort (minimum number of species recorded in "surveyed squares")				level of recording effort (minimum number of species recorded in "surveyed squares")			
		1	2	3	4	1	2	3	4
Conocephalus discolor	G	3.25	3.38	3.42	3.47	3.20	3.33	3.35	3.41
	p	0.0018	0.0007	0.0005	0.0003	0.0025	0.0010	0.0009	0.0006
Metrioptera roeselii	G	3.43	3.38	3.34	3.33	3.32	3.26	3.20	3.19
	p	0.0004	0.0005	0.0007	0.0008	0.0009	0.0014	0.0021	0.0022

Test statistics (G) and p-values (p) for "uncorrected" and "corrected range change" and four levels of recording effort. In each case, *Conocephalus discolor* and *Metrioptera roeselii* were identified as outliers.
