# Supplementary material for: Two Species with an Unusual Combination of Traits Dominate Responses of British Grasshoppers and Crickets to Environmental Change
Source: PLoS One. 2015 Jun 25;10(6):e0130488. doi: 10.1371/journal.pone.0130488 (PMC4482502; doi:10.1371/journal.pone.0130488)
Supplement: S3 Table — (PDF) [file pone.0130488.s006.pdf]

S3 Table. Results of Shapiro-Wilk tests for normality of residuals.

			test statistic W			p-value			
	range change measure	recording effort level	min	median	max	min	median	max	% of p-values >= 0.05
models with all species	"uncorrected"	1	0.906	0.957	0.974	0.033	0.405	0.790	98
		2	0.929	0.961	0.982	0.104	0.480	0.935	100
		3	0.918	0.962	0.985	0.060	0.506	0.974	100
		4	0.932	0.968	0.985	0.122	0.631	0.975	100
	"corrected"	1	0.913	0.958	0.974	0.048	0.418	0.789	98
		2	0.921	0.961	0.977	0.069	0.488	0.844	100
		3	0.919	0.960	0.983	0.063	0.468	0.945	100
		4	0.936	0.960	0.980	0.147	0.454	0.911	100
models with species excluding *C. discolor* and *M. roeselii*	"uncorrected"	1	0.855	0.924	0.977	0.005	0.104	0.871	81
		2	0.881	0.949	0.978	0.016	0.327	0.890	96
		3	0.907	0.944	0.978	0.048	0.266	0.897	98
		4	0.938	0.967	0.987	0.195	0.671	0.988	100
	"corrected"	1	0.863	0.924	0.978	0.007	0.104	0.896	91
		2	0.895	0.955	0.978	0.028	0.420	0.889	98
		3	0.909	0.949	0.980	0.053	0.320	0.921	100
		4	0.942	0.971	0.991	0.239	0.764	0.998	100

Minima, medians and maxima of Shapiro-Wilk test statistic W and associated p-values, testing for normality of residuals of top GLM model sets with ΔAIC<4 for two range change measures and four levels of recording effort. Results for models with all species (top half of table) and models with species excluding *Conocephalus discolor* and *Metrioptera roeselii* (bottom half).
