# Supplementary material for: Two Species with an Unusual Combination of Traits Dominate Responses of British Grasshoppers and Crickets to Environmental Change
Source: PLoS One. 2015 Jun 25;10(6):e0130488. doi: 10.1371/journal.pone.0130488 (PMC4482502; doi:10.1371/journal.pone.0130488)
Supplement: S4 Table — (PDF) [file pone.0130488.s007.pdf]

S4 Table. Moran's I phylogenetic autocorrelation indices and associated p-values.

	range change measure	recording effort level	Moran's I min	Moran's I median	Moran's I max	p-value min	p-value median	p-value max	% of p-values >= 0.05
models with all species	"uncorrected"	1	0.084	0.112	0.132	0.040	0.066	0.132	96
		2	0.086	0.112	0.132	0.040	0.067	0.126	96
		3	0.084	0.102	0.174	0.008	0.086	0.131	98
		4	0.086	0.099	0.116	0.063	0.092	0.122	100
	"corrected"	1	0.075	0.109	0.129	0.044	0.073	0.158	98
		2	0.073	0.110	0.129	0.041	0.071	0.164	95
		3	0.080	0.099	0.119	0.046	0.093	0.141	98
		4	0.081	0.097	0.112	0.054	0.098	0.138	100
models with species excluding *C. discolor* and *M. roeselii*	"uncorrected"	1	0.087	0.126	0.194	0.010	0.055	0.131	56
		2	0.102	0.131	0.200	0.007	0.049	0.096	46
		3	0.101	0.129	0.201	0.007	0.054	0.096	58
		4	0.103	0.133	0.209	0.006	0.051	0.099	51
	"corrected"	1	0.088	0.122	0.186	0.013	0.060	0.132	65
		2	0.105	0.129	0.193	0.009	0.053	0.090	58
		3	0.103	0.131	0.198	0.009	0.051	0.094	50
		4	0.109	0.128	0.206	0.007	0.058	0.090	65

Minima, medians and maxima for top GLM model sets with $\Delta AIC<4$ for two range change measures and four levels of recording effort. Results for models with all species (top half of table) and models with species excluding *Conocephalus discolor* and *Metrioptera roeselii* (bottom half).
