# Supplementary material for: Two Species with an Unusual Combination of Traits Dominate Responses of British Grasshoppers and Crickets to Environmental Change
Source: PLoS One. 2015 Jun 25;10(6):e0130488. doi: 10.1371/journal.pone.0130488 (PMC4482502; doi:10.1371/journal.pone.0130488)
Supplement: S5 Table — (PDF) [file pone.0130488.s008.pdf]

S5 Table. Impacts of species traits on distribution changes of British grasshoppers and crickets (all species) between the 1980s and 2000s, phylogenetic models.

	trait	"uncorrected range change"				"corrected range change"			
		% included	weighted mean coefficient	weighted mean standard error	significance	% included	weighted mean coefficient	weighted mean standard error	significance
(Intercept)		100	39.09	16.36	*	100	45.17	22.48	*
habitat and resource use	(i) breadth of habitat use	100	1.38	0.48	**	100	1.95	0.64	**
	(ii) vegetation structure:	45				45			
	short vs. medium		-0.25	0.38	n.s.		-0.35	0.53	n.s.
	short vs. tall		0.49	0.55	n.s.		0.56	0.73	n.s.
	medium vs. tall		0.74	0.44	n.s.		0.91	0.57	n.s.
	(iii) oviposition site:	100				100			
	vegetation vs. ground		1.07	0.38	**		1.47	0.51	**
	vegetation vs. ground or vegetation		0.98	0.42	*		1.35	0.58	*
	ground vs. ground or vegetation		-0.09	0.29	n.s.		-0.12	0.39	n.s.
	(iv) diet:	21				26			
	herbivorous vs. not herbivorous		-0.08	0.35	n.s.		-0.18	0.47	n.s.
life history	(v) mean body size	26	-0.50	1.29	n.s.	26	-0.26	1.78	n.s.
	(vi) generations per year:	11				9			
	one vs. half		-0.32	0.48	n.s.		-0.45	0.64	n.s.
	one vs. half or one		-0.21	0.67	n.s.		-0.25	0.88	n.s.
	half vs. half or one		0.12	0.62	n.s.		0.20	0.82	n.s.
	(vii) winter stage:	28				30			
	egg vs. not egg		0.01	0.63	n.s.		0.13	0.91	n.s.
	(viii) phenology	30	-0.17	0.21	n.s.	38	-0.26	0.29	n.s.
dispersal ability	(ix) wing morph:	21				19			
	short vs. long		0.12	0.78	n.s.		0.22	1.06	n.s.
	short vs. dimorphic		-0.14	0.92	n.s.		-0.18	1.19	n.s.
	long vs. dimorphic		-0.27	0.42	n.s.		-0.40	0.53	n.s.
	(x) wing load	40	0.32	0.32	n.s.	38	0.39	0.46	n.s.
distribution	(xi) average latitude	100	-0.76	0.32	*	98	-0.90	0.43	*

Summary of results for sets of top PGLS models with ΔAIC<4 (47 models for "uncorrected range change", and 53 models for "corrected range change"). The importance of traits is indicated by the frequency with which they are included in the top model set (% included), and by their weighted mean coefficients, standard errors and significance levels. Significance levels: *= p<0.05, **=p<0.01. Results given are for minimum adequate recording effort, i.e. for "surveyed squares" with a minimum of 1 species recorded in both 1980-9 and 2000-9.
