# Supplementary material for: Two Species with an Unusual Combination of Traits Dominate Responses of British Grasshoppers and Crickets to Environmental Change
Source: PLoS One. 2015 Jun 25;10(6):e0130488. doi: 10.1371/journal.pone.0130488 (PMC4482502; doi:10.1371/journal.pone.0130488)
Supplement: S6 Table — (PDF) [file pone.0130488.s009.pdf]

S6 Table. Amount of overall variation explained by models.

		"uncorrected range change"				"corrected range change"			
		level of recording effort (minimum number of species recorded in "surveyed squares")				level of recording effort (minimum number of species recorded in "surveyed squares")			
		1	2	3	4	1	2	3	4
all species	minimum	0.033	0.033	0.038	0.032	0.029	0.031	0.033	0.030
	weighted mean	0.543	0.515	0.495	0.506	0.558	0.530	0.519	0.525
	maximum	0.590	0.569	0.554	0.563	0.607	0.586	0.578	0.582
species excluding *C. discolor* and *M. roeselii*	minimum	0.000	0.002	0.004	0.000	0.000	0.001	0.007	0.032
	weighted mean	0.118	0.126	0.131	0.129	0.116	0.130	0.137	0.167
	maximum	0.241	0.192	0.194	0.196	0.237	0.189	0.198	0.242

Minima, weighted means and maxima of adjusted deviance (D^2) for top sets of GLM models for two range change measures and four levels of recording effort.
