# Supplementary material for: Two Species with an Unusual Combination of Traits Dominate Responses of British Grasshoppers and Crickets to Environmental Change
Source: PLoS One. 2015 Jun 25;10(6):e0130488. doi: 10.1371/journal.pone.0130488 (PMC4482502; doi:10.1371/journal.pone.0130488)
Supplement: S8 Table — (PDF) [file pone.0130488.s011.pdf]

S8 Table. Fitted range change values.

	all species								species excluding *C. discolor* and *M. roeselii*							
	"uncorrected range change" recording effort level				"corrected range change" recording effort level				"uncorrected range change" recording effort level				"corrected range change" recording effort level			
**Species**	**1**	**2**	**3**	**4**	**1**	**2**	**3**	**4**	**1**	**2**	**3**	**4**	**1**	**2**	**3**	**4**
M. thalassinum	0.33±0.24	0.29±0.25	0.25±0.27	0.32±0.25	0.25±0.29	0.2±0.3	0.2±0.31	0.27±0.3	0.08±0.09	0.05±0.08	0.03±0.08	0.04±0.11	0.21±0.23	0.16±0.21	0.1±0.2	0.14±0.32
T. viridissima	-0.04±0.09	-0.04±0.09	-0.04±0.1	-0.05±0.08	-0.42±0.12	-0.42±0.12	-0.42±0.13	-0.42±0.1	-0.22±0.13	-0.26±0.13	-0.28±0.13	-0.31±0.13	-0.71±0.39	-0.82±0.35	-0.8±0.35	-0.93±0.35
P. griseoaptera	0.48±0.15	0.54±0.16	0.53±0.17	0.66±0.16	0.49±0.2	0.57±0.21	0.61±0.21	0.74±0.19	0.07±0.07	0.06±0.07	0.03±0.08	0.13±0.12	0.1±0.18	0.06±0.17	0.07±0.2	0.3±0.31
P. albopunctata	0.08±0.05	0.06±0.05	0.08±0.05	0.04±0.05	-0.34±0.09	-0.36±0.1	-0.38±0.09	-0.4±0.07	-0.08±0.06	-0.09±0.04	-0.1±0.04	-0.1±0.05	-0.22±0.17	-0.25±0.12	-0.26±0.1	-0.28±0.11
M. brachyptera	-0.03±0.13	-0.01±0.14	-0.03±0.15	0±0.15	-0.35±0.18	-0.34±0.2	-0.38±0.2	-0.36±0.18	-0.01±0.13	0.04±0.09	0.02±0.08	0.05±0.09	-0.03±0.31	0.1±0.22	0.06±0.22	0.14±0.23
M. roeselii	1.11±0.14	1.11±0.15	1.08±0.16	1.1±0.16	1.26±0.2	1.22±0.2	1.17±0.2	1.16±0.19	-	-	-	-	-	-	-	-
C. discolor	1.93±0.08	1.96±0.09	1.99±0.11	2.01±0.1	2.26±0.12	2.25±0.17	2.22±0.17	2.23±0.16	-	-	-	-	-	-	-	-
C. dorsalis	1.16±0.1	1.15±0.12	1.14±0.12	1.14±0.12	1.28±0.14	1.22±0.16	1.17±0.15	1.16±0.15	0.16±0.11	0.11±0.08	0.07±0.08	0.09±0.09	0.38±0.31	0.26±0.24	0.19±0.22	0.26±0.27
L. punctatissima	0.35±0.15	0.4±0.15	0.41±0.16	0.55±0.15	0.27±0.2	0.34±0.2	0.39±0.2	0.55±0.17	0.14±0.09	0.15±0.09	0.13±0.1	0.26±0.12	0.35±0.25	0.38±0.24	0.36±0.26	0.71±0.32
N. sylvestris	-0.41±0.15	-0.35±0.16	-0.31±0.19	-0.29±0.17	-0.91±0.2	-0.81±0.21	-0.76±0.22	-0.7±0.2	0.14±0.13	0.23±0.1	0.26±0.09	0.29±0.1	0.52±0.33	0.73±0.28	0.77±0.25	0.88±0.23
T. ceperoi	0.57±0.09	0.57±0.09	0.61±0.13	0.56±0.08	0.36±0.12	0.35±0.13	0.35±0.16	0.32±0.12	0.17±0.1	0.17±0.11	0.2±0.1	0.15±0.11	0.53±0.25	0.55±0.24	0.59±0.23	0.47±0.28
T. subulata	0.3±0.08	0.29±0.08	0.32±0.11	0.24±0.08	0.15±0.1	0.12±0.11	0.16±0.12	0.05±0.11	0.15±0.06	0.16±0.06	0.17±0.06	0.12±0.06	0.37±0.18	0.4±0.17	0.47±0.15	0.29±0.16
T. undulata	0.31±0.08	0.29±0.09	0.3±0.11	0.22±0.1	0.2±0.11	0.18±0.13	0.21±0.14	0.09±0.13	0.18±0.07	0.19±0.07	0.2±0.06	0.14±0.07	0.43±0.2	0.48±0.2	0.56±0.16	0.36±0.19
S. grossum	-0.18±0.06	-0.22±0.08	-0.19±0.08	-0.22±0.09	-0.66±0.09	-0.72±0.13	-0.7±0.12	-0.72±0.12	-0.15±0.1	-0.19±0.09	-0.19±0.09	-0.19±0.08	-0.45±0.27	-0.57±0.25	-0.55±0.24	-0.56±0.21
S. lineatus	-0.12±0.1	-0.13±0.11	-0.12±0.11	-0.13±0.11	-0.46±0.14	-0.44±0.17	-0.42±0.15	-0.44±0.14	-0.09±0.07	-0.11±0.05	-0.12±0.05	-0.12±0.05	-0.33±0.21	-0.36±0.17	-0.34±0.14	-0.4±0.15
O. rufipes	-0.61±0.13	-0.53±0.16	-0.53±0.16	-0.46±0.17	-1.24±0.2	-1.1±0.23	-1.12±0.22	-1.01±0.23	-0.19±0.15	-0.09±0.09	-0.08±0.07	-0.07±0.06	-0.48±0.42	-0.21±0.21	-0.22±0.2	-0.2±0.17
O. viridulus	-0.25±0.09	-0.32±0.08	-0.39±0.08	-0.42±0.08	-0.49±0.14	-0.56±0.14	-0.57±0.13	-0.67±0.12	-0.03±0.07	-0.06±0.05	-0.09±0.05	-0.1±0.05	-0.21±0.2	-0.27±0.16	-0.26±0.15	-0.35±0.17
C. brunneus	-0.28±0.07	-0.26±0.07	-0.3±0.06	-0.28±0.07	-0.56±0.09	-0.51±0.1	-0.51±0.1	-0.49±0.1	-0.09±0.08	-0.09±0.06	-0.12±0.06	-0.13±0.06	-0.36±0.22	-0.34±0.17	-0.34±0.16	-0.43±0.16
C. vagans	0.14±0.05	0.17±0.06	0.19±0.06	0.21±0.06	-0.25±0.08	-0.21±0.09	-0.22±0.09	-0.16±0.08	-0.05±0.06	-0.04±0.05	-0.04±0.05	-0.03±0.06	-0.1±0.18	-0.07±0.13	-0.11±0.13	-0.04±0.17
C. parallelus	0.42±0.08	0.42±0.09	0.4±0.15	0.37±0.08	0.37±0.12	0.37±0.12	0.36±0.17	0.3±0.11	0.03±0.11	-0.01±0.1	-0.05±0.09	-0.05±0.09	-0.09±0.27	-0.17±0.2	-0.17±0.2	-0.23±0.22
C. albomarginatus	-0.07±0.07	-0.15±0.08	-0.17±0.08	-0.23±0.09	-0.36±0.1	-0.46±0.13	-0.47±0.13	-0.56±0.14	0.02±0.06	-0.03±0.04	-0.05±0.04	-0.05±0.04	-0.01±0.18	-0.12±0.1	-0.14±0.1	-0.13±0.11
G. rufus	0.04±0.08	0.02±0.1	0.02±0.11	0.01±0.12	-0.3±0.13	-0.34±0.18	-0.34±0.17	-0.33±0.18	-0.02±0.09	-0.05±0.06	-0.06±0.05	-0.05±0.07	-0.06±0.26	-0.14±0.16	-0.18±0.15	-0.12±0.19
M. maculatus	-0.44±0.06	-0.44±0.06	-0.5±0.06	-0.49±0.06	-0.76±0.09	-0.74±0.1	-0.74±0.1	-0.75±0.1	-0.01±0.08	0.01±0.07	-0.01±0.07	-0.01±0.07	-0.07±0.22	0.02±0.17	-0.01±0.19	-0.05±0.18

Weighted means ± weighted standard deviations across sets of top GLM models with ΔAIC<4 (weightings by Akaike weights). Results for four levels of recording effort, "uncorrected" and "corrected range change", and for models with all species and models excluding *C. discolor* and *M. roeselii*
